# Regulation of ULK1 by WTAP/IGF2BP3 axis enhances mitophagy and progression in epithelial ovarian cancer

**DOI:** 10.1038/s41419-024-06477-0

**Published:** 2024-01-29

**Authors:** Jiao Wang, Fei Zheng, Dandan Wang, Qing Yang

**Affiliations:** Department of Obstetrics and Gynecology, Shengjing Hospital of China Medical University, Shenyang, 110004 China

**Keywords:** Ovarian cancer, Mitophagy

## Abstract

There is a pressing need for innovative therapeutic strategies for patients with epithelial ovarian cancer (EOC). Previous studies have shown that UNC-51-like kinase 1 (ULK1), a serine/threonine kinase, is crucial in regulating cellular autophagy and mitophagy across various tumor types. However, the clinical implications, biological functions, and potential mechanisms of ULK1 in EOC remain poorly understood. This study demonstrates that ULK1 expression is upregulated in EOC tissue samples and EOC cell lines, with increased ULK1 expression correlating with poor prognosis. Functionally, overexpressed ULK1 enhances the proliferation and migration abilities of EOC cells both in vitro and in vivo. Mechanistically, ULK1 was identified as an m6A target of WTAP. WTAP-mediated m6A modification of ULK1 enhanced its mRNA stability in an IGF2BP3-dependent manner, leading to elevated ULK1 expression and enhanced mitophagy in EOC. In summary, our research reveals that the WTAP/IGF2BP3-ULK1 axis significantly influences protective mitophagy in EOC, contributing to its progression. Therefore, the regulatory mechanisms and biological function of ULK1 identify it as a potential molecular target for therapeutic intervention in EOC.

## Introduction

Epithelial ovarian cancer (EOC) is marked by a high fatality rate and a poorer prognosis compared to other gynecological malignancies. Despite advances in diagnostic techniques, surgical procedures, and the development of novel therapeutic agents, long-term survival rates for EOC patients remain unsatisfactory. Patients often face relapse and chemoresistance within a few years, especially in advanced stages of the disease [1, 2]. These are significant factors contributing to an unfavorable prognosis. Consequently, identifying novel therapeutic targets and potential tumor prognostic markers is of urgent necessity.

Mitophagy, the process in which dysfunctional or superfluous mitochondria are selectively engulfed by autophagosomes and then degraded in lysosomes, is recognized as a crucial mechanism for mitochondrial quality control [3]. As a selective form of autophagy, it aids in the removal of impaired mitochondria and the maintenance of the balance of the mitochondrial environment, thus maintaining cellular homeostasis [4]. Aberrant mitophagy has been associated with various diseases, including nervous system disorders [5], acute and chronic kidney diseases [6, 7], cardiovascular diseases [8], and cancer [9, 10]. In mammals, two primary molecular regulatory mechanisms govern mitophagy [11]. The first is the Parkin-dependent pathway, involving mitophagy mediated by PINK1/Parkin. The second is the Parkin-independent pathway, where mitophagy is primarily regulated by receptor proteins like BNIP3, BNIP3L/NIX, and FUNDC1 [10]. The complex impacts of mitophagy on cancer cells, and its specific role in EOC, are still largely unexplored.

In our previous study, we analyzed the mutational status of mitophagy-related genes in samples from The Cancer Genome Atlas (TCGA) ovarian cancer (OV) dataset. Our findings indicated that 4.36% of the samples had gene mutations. Additionally, a significant prevalence of copy number variations (CNV) was observed in mitophagy-related genes [12]. This suggests that alterations in mitophagy may play a role in the onset and progression of OV. UNC-51-like kinase 1 (ULK1), a serine/threonine kinase, shares homology with autophagy-related protein (ATG) 1 in yeast and UNC-51 in Caenorhabditis elegans [13]. It acts as a catalytic component of the autophagy activation complex and is known to specifically regulate mitophagy [14–17]. ULK1 has been extensively documented in various human diseases, including cancer [13]. Our previous bioinformatics analysis revealed that ULK1 is a significant prognostic factor in OV, with high expression levels correlating with a decreased overall survival (OS) period [12]. However, the specific role and molecular regulatory mechanism of ULK1 in the progression of EOC remain unclear.

The N6-methyladenosine (m6A) modification of RNA involves methylation at the 6th nitrogen atom of adenine, catalyzed by the methyltransferase complex (MTC; writer). The modified recognition protein (reader) specifically recognizes the m6A site, playing a key role in post-transcriptional RNA regulation. This affects RNA splicing, localization, stability, degradation, and translation [18, 19]. The demethylation of m6A is facilitated by demethylase (eraser), creating a dynamic regulatory network for m6A methylation modification. Recent studies have highlighted m6A modification’s significant role in various cancers, including OV [20–24]. Bioinformatics analysis has indicated multiple m6A modification sites on ULK1 mRNA, suggesting its potential importance in ULK1 expression regulation.

In this study, we report on the oncogenic roles of ULK1 in EOC by evaluating its clinical significance in EOC patients and examining its effects on mitophagy and the malignant behaviors of EOC cells both in vitro and in vivo. Additionally, we further demonstrate that ULK1 is regulated at the post-transcriptional level by the m6A writer Wilms’ tumor 1-associating protein (WTAP) and reader insulin-like growth factor 2 mRNA binding protein 3 (IGF2BP3). Our research is significant in identifying potential prognostic markers and therapeutic targets for EOC.

## Materials and methods

### Human tissue specimens

This study received approval from the Scientific Research and New Technology Ethical Committee of Shengjing Hospital of China Medical University. Written informed consent was obtained from all patients prior to the commencement of the study. Clinical specimens were collected from surgically resected tissues of patients, accompanied by detailed clinical characteristics and extensive long-term follow-up data, spanning from January 2016 to December 2018 at Shengjing Hospital of China Medical University. None of the patients with EOC had undergone chemotherapy, radiotherapy, or other antitumor therapies before surgery. To assess ULK1 expression, immunohistochemistry (IHC) was performed on paraffin-embedded sections from 5 normal ovarian tissues, 6 benign ovarian tumor tissues, 14 borderline ovarian tumor tissues, and 78 EOC tissues.

### Cell culture

The human EOC cell lines OVCAR-3, Caov-3, ES-2 and A2780 were purchased from the Chinese Academy of Sciences Cell Bank (Shanghai, China). The human normal ovarian epithelial cell line HOSEpiC were purchased from the American Type Culture Collection (ATCC, USA). OVCAR-3, Caov-3, A2780, and HOSEpiC were cultured in RPMI 1640 medium (Procell, Wuhan, China), supplemented with 10% fetal bovine serum (FBS; Procell). ES-2 was cultured in McCoy’s 5 A medium (Procell), also supplemented with 10% FBS. All cells were maintained under controlled conditions at a temperature of 37°C in an atmosphere containing 5% CO_2_. Regular monitoring for mycoplasma contamination was performed using PCR.

### Western blot

Protein extraction was conducted using Radioimmunoprecipitation assay (RIPA) lysis buffer (Beyotime, Shanghai, China). The supernatant’s protein concentration was determined using the BCA technique, followed by denaturation through the addition of 5× loading buffer and heating at 100 °C for 5 min. Proteins were then separated by 10% or 12.5% sodium dodecyl sulfate-polyacrylamide gel electrophoresis and subsequently transferred onto a 0.22 µm PVDF membrane. This membrane was blocked with a 5% non-fat milk solution for 2 h at room temperature (RT) and incubated with primary antibodies overnight at 4 °C. The following day, the membranes were incubated with secondary antibodies at RT for 1 h. Protein bands were visualized using enhanced chemiluminescence (Thermo Scientific, Carlsbad, CA, USA) via Image Lab software (Bio-Rad, CA, USA). The primary antibodies utilized for the western blot are listed in Table S1. The original western blots were shown in Supplementary Material 8.

### Reverse transcription-quantitative polymerase chain reaction (RT-qPCR)

Total RNA extraction was performed using TRIzol reagent (Takara Bio, Kusatsu, Japan), with RNA concentration and purity assessed using a NanoDrop 2000 system (Thermo Scientific). The reverse transcription of RNA samples and the qPCR analysis were carried out in accordance with our previously documented methodology [12]. The 2^−∆∆Ct^ method was employed for gene expression analysis, using ACTB as the internal reference. The primer sequences utilized for RT-qPCR can be found in Supplementary Table S2.

### IHC staining

Tissues were fixed in 10% neutral formalin and then processed into paraffin-embedded sections, each 4 µm thick. These sections underwent deparaffinization, hydration, and immersion in 3% H_2_O_2_ at RT for 20 min. Following this, they were incubated overnight with ULK1-specific antibodies (20986-1-AP, Proteintech, 1:300) or Ki-67 antibodies (9027 T, Cell Signaling Technology, 1:800) at 4 °C. On the subsequent day, the sections were treated with biotinylated goat anti-rabbit antibodies for 1 h, stained with diaminobenzidine (DAB; Maixin Biotechnology, Fuzhou, China), and then counterstained with hematoxylin (Maixin Biotechnology). Two independent pathologists, blind to the experimental data, evaluated the specimens. Scoring was based on the percentage of positively stained cells (0 = 0–4%, 1 = 5–25%, 2 = 26–50%, 3 = 51–75%, 4 = 76–100%) and staining intensity (0 = none, 1 = slight, 2 = moderate, 3 = strong). The total score for each visual field was the product of the percentage and intensity scores. The IHC score was calculated as previously reported [25].

### Cell transfection and lentiviral infection

Small interfering RNAs (siRNAs) targeting human ULK1, WTAP, METTL3, METTL14, ALKBH5, FTO, IGF2BP1/2/3, and their respective negative controls were acquired from GenePharma (Suzhou, China) and Jintuosi (Wuhan, China). These siRNAs were transfected into cells using the transfection reagent GP-transfect-Mate (GenePharma), following the manufacturer’s instructions. The sequences of these siRNAs are detailed in Supplementary Table S3. Lentiviruses designed to overexpress ULK1 or WTAP, or to express ULK1 or WTAP short hairpin RNA (shRNA), along with their corresponding negative control vectors, were sourced from GeneChem (Shanghai, China). The OVCAR-3, A2780, and ES-2 cell lines were infected with these lentiviruses at multiplicity of infection (MOI) levels of 30, 10, and 5, respectively.

### Cell viability assay

Cell viability was evaluated using the CCK-8 kit (GK10001, GLPBIO, Montclair, CA, USA). Cells were distributed in a 96-well plate with a density of 2 × 10^3^ cells per well. Every 24 h, 10 μL of CCK-8 solution was added to each well. Following a 2-h incubation period, the absorbance at 450 nm was quantified utilizing a microplate reader.

### Colony formation assay

1000 cells were seeded into each well of 6-well plates. After a 10-day culture period, the cells were subjected to fixation using a 4% paraformaldehyde (PFA) solution, followed by staining with a 0.5% crystal violet solution. Colonies, defined as aggregations of more than 50 cells, were counted.

### Cell scratch assay

Cells were seeded and cultured in 6-well plates until they achieved 80–90% confluency. Then, a straight line scratch was carefully made in each well using a 200 µL pipette tip, followed by rinsing with phosphate-buffered saline (PBS). The created scratch area was imaged using a microscope (Nikon, Japan). Before retaking the images, the cells were incubated in a serum-free medium for 24 h.

### Transwell migration assay

The cell migration assay was conducted by employing a Transwell chamber equipped with an 8-μm pore size filter, inserted into a 24-well plate. The lower chamber was added with 700 μL of medium supplemented with 10% FBS, while the upper chamber was seeded with cells suspended in FBS-free medium at a density of 2 × 10^4^ cells/200 μL. Following a 24-h incubation period, the cells were fixed with 4% PFA and stained with 0.5% crystal violet. The migrated cells were then photographed and quantified.

### Apoptosis assay with flow cytometry

Cells were collected and processed into a single-cell suspension. Annexin V-FITC and PI staining solution (Vazyme, Nanjing, China) were applied in accordance with the instructions provided by the manufacturer. The cells were then incubated for 10 min at RT, shielded from light. Flow cytometry (Beckman Coulter, Brea, CA, USA) was utilized for subsequent analysis.

### Mitotracker staining

Seed cells in confocal dishes until they reach the appropriate density. Prepare the MitoTracker Red CMXRos (Beyotime) working solution according to the manufacturer’s instructions. Remove the cell culture medium and add the MitoTracker Red CMXRos working solution. Incubate for 30 min at 37 °C. After incubation, remove the MitoTracker Red CMXRos solution and add fresh culture medium, maintained at 37 °C, containing 10% FBS. Subsequently, perform immunofluorescence (IF) staining following cell fixation and permeabilization.

### IF staining

Cells were fixed with 4% PFA for 30 min and permeabilized with 0.3% Triton X-100 for 15 min at RT. Following the washing step with PBS, the cells underwent blocking by exposure to 10% goat serum at RT for 30 min. They were then incubated at 4 °C overnight with anti-LC3 antibody (1:300, 14600-1-AP, Proteintech). The following day, after PBS washing, cells were incubated with a fluorescence-conjugated secondary antibody at RT for 1 h. Subsequently, cell nuclei were stained with DAPI (Solarbio, Beijing, China) for 10 min. The acquisition of images was performed utilizing a laser confocal microscope (LSM880, Zeiss, Jena, Germany).

### Transmission electron microscopy (TEM)

Cell samples were pre-fixed in 2.5% glutaraldehyde at 4 °C for 2 h. Following this, they were washed with sodium dimethyl arsenate buffer and then post-fixed in 1% osmium tetroxide at 4 °C for 2 h. After additional washing with ddH_2_O, the samples underwent a graded dehydration process in ethanol solutions of increasing concentrations: 30%, 50%, 70%, followed by 80%, 90%, and two changes of 100% acetone, each step lasting 10–15 min. The samples were then infiltrated in a 1:1 mixture of epoxy resin and acetone at RT overnight. The dehydrated cells were subsequently embedded in epoxy resin, undergoing a polymerization process at 35 °C for 24 h, 45 °C for 24 h, and finally 60 °C for 24 h. Sections with a thickness of 60 nm were meticulously prepared, subsequently subjected to a 10-min staining process using uranyl acetate, and then further stained with lead citrate for 5 min. After air drying, images were captured using a TEM (JEM-1400Flash, JEOL; Tokyo, Japan).

### Animal study

The animal research received approval from the Scientific Research and New Technology Ethical Committee of Shengjing Hospital of China Medical University (Reference No. 2021PS410K). A xenograft mouse model was developed using 5-week-old female BALB/c nude mice, sourced from HFK Bioscience, Changping, Beijing, China. These mice had continuous access to water and food and were housed in a specific pathogen-free (SPF) environment. The nude mice were randomly assigned to groups (*n* = 6/group), and each received a subcutaneous injection of 1 × 10^6^ ES-2 cells into the right axilla. The measurement of tumor size was conducted every 3 days, wherein the calculation of tumor volumes was performed using the formula: tumor volume (mm^3^) = length × width^2^/2. The mice were euthanized 21 days post-injection, and their transplanted tumors were harvested for subsequent analysis.

### Bioinformatic analyses

The Sequence-based RNA Adenosine Methylation Site Predictor (SRAMP) (https://www.cuilab.cn/sramp/) is a valuable and freely available tool. It utilizes a random forest machine-learning framework to predict m6A modification sites based on sequence-derived features [26]. Using the full transcript mode of SRAMP, the m6A locus was predicted in human ULK1 pre-mRNA. The GEPIA database (http://gepia.cancer-pku.cn/) [27] was employed for analyzing IGF2BP3 mRNA expression in normal ovarian tissues and OV. The Kaplan–Meier plotter database (http://kmplot.com/) [28] was used to evaluate the prognostic significance of ULK1 and IGF2BP3 in OV. Additionally, RM2Target (http://rm2target.canceromics.org/) [29] facilitated the investigation of the correlation between the expressions of IGF2BP3 and ULK1 in TCGA cancers, including the associations between IGF2BP3 and ULK1. The data of mutations were downloaded and visualized as previously described [12]. The ggplot2 package in R software was used to present the expression distribution of m6A-related genes. The LinkedOmics database (http://www.linkedomics.org/login.php) [30] was used to analyze differentially expressed genes in *TP53* mutant and *TP53* wild-type OV.

### RNA total m6A quantification

The total m6A level in treated cells was quantified using the EpiQuik m6A RNA Methylation Quantification Kit (P-9005, Epigentek, NY, USA), following the manufacturer’s specifications. The quantification of m6A content was performed through the measurement of absorbance at 450 nm.

### RNA immunoprecipitation (RIP)

The RNA immunoprecipitation (RIP) assay was conducted using a RIP kit (Bes5101, BersinBio, Guangzhou, China) and anti-WTAP (41934 S, Cell Signaling Technology) or anti-IGF2BP3 (14642-1-AP, Proteintech) antibodies, following the manufacturer’s protocol. IgG served as a control. RT-qPCR was carried out as previously described.

### m6A methylated RNA immunoprecipitation (MeRIP)

The MeRIP assay was conducted following the manufacturer’s protocol of the EpiQuik CUT & RUN m6A RNA Enrichment Kit (P-9018, Epigentek). This process involved selective isolation of m6A-containing fragments using beads-bound m6A capture antibody. The Cleavage Enzyme Mix was employed to cleave the RNA sequences located at the termini of the m6A-containing regions. The enriched RNA was subsequently released, purified, and eluted. The quantification of alterations in m6A methylation of the target gene was performed using RT-qPCR.

### RNA stability assay

Upon reaching 60–80% confluency, cultured cells were treated with actinomycin D (ACD, catalog number GC16866, from GLPBIO) at a concentration of 10 µg/mL. Following the incubation with ACD, cellular RNA was extracted at 0, 3, and 6 h for subsequent analysis using RT-qPCR.

### Statistical analysis

Statistical analyses were conducted using GraphPad Prism 9 software (La Jolla, CA, USA) and R (v4.0.3). Data are presented in the form of a mean ± SEM based on a minimum of three independent experiments. The correlation between the expression levels of ULK1 and the clinicopathological characteristics of patients was analyzed using the chi-squared test. The unpaired Student’s *t*-test was utilized for conducting comparisons between two groups. Analysis of variance (ANOVA) was utilized for comparing three or more groups. Statistical significance was established at *p* < 0.05, with specific levels indicated as *p < 0.05, **p < 0.01, ***p < 0.001, and ****p < 0.0001.

## Results

### ULK1 expression is elevated in EOC, associating high ULK1 expression with EOC progression

**Fig. 1:**
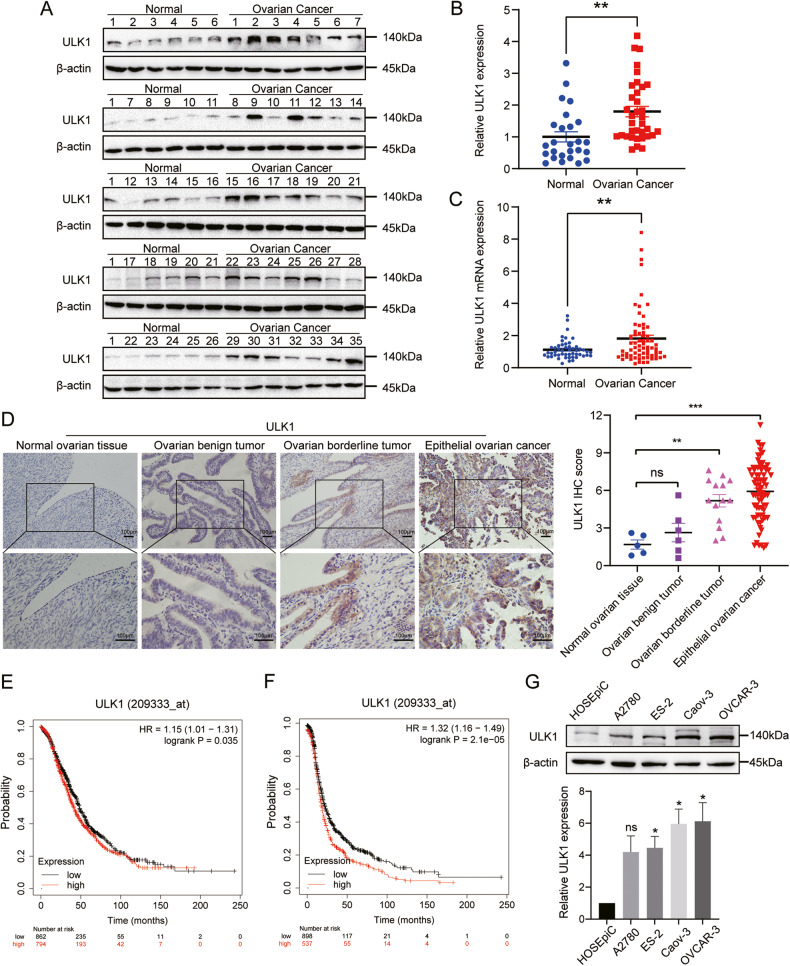
ULK1 is overexpressed in EOC, and its elevated expression is associated with poor prognosis in EOC patients. **A**, **B** Protein expression levels of ULK1 in 35 EOC and 26 normal ovarian tissues were detected by western blot. **C** The mRNA expression levels of ULK1 in 67 EOC and 50 normal ovarian tissues were detected using RT-qPCR. **D** Representative images of ULK1 IHC staining in EOC (*n* = 78), epithelial ovarian borderline tumor (*n* = 14), epithelial ovarian benign tumor (*n* = 6), and normal ovarian tissues (*n* = 5). **E**, **F** Kaplan–Meier plots for the OS and PFS of OV patients according to ULK1 expression. **G** The expression levels of ULK1 protein in human EOC cell lines A2780, ES-2, Caov-3, and OVCAR-3, and human normal ovarian epithelial cell line HOSEpiC were detected by western blot. ns not significant; *P < 0.05; **P < 0.01; ***P < 0.001.

Our prior bioinformatics analysis suggested ULK1 as a prognostic risk factor for patients in the TCGA-OV dataset, with its high expression correlating to shortened OS periods [12]. To examine ULK1 expression in EOC, we initially analyzed the protein expression of ULK1 in 35 primary EOC tissues and 26 normal ovarian tissues using western blot assays, and the mRNA expression in 67 primary EOC tissues and 50 normal ovarian tissues via RT-qPCR assays. We observed higher expression levels of ULK1 in primary EOC tissues than in normal ovarian tissues at both protein (Fig. 1A, B) and mRNA (Fig. 1C) levels. We then investigated ULK1 expression in 78 primary EOC specimens, 14 epithelial ovarian borderline tumor specimens, 6 epithelial ovarian benign tumor specimens, and 5 normal ovarian specimens through IHC assays. Compared to normal ovarian tissue, protein levels of ULK1 were elevated in primary EOC and epithelial ovarian borderline tumor tissues, with no significant change observed in benign ovarian tumors (Fig. 1D). A comprehensive analysis of ULK1 expression in relation to clinicopathological parameters was also conducted (Table 1). Statistical analyses indicated a positive correlation between upregulated ULK1 expression and higher International Federation of Gynecology and Obstetrics (FIGO) stages. Consistently, Kaplan–Meier analysis revealed that patients with increased ULK1 levels had less favorable OS (Fig. 1E) and progression-free survival (PFS) (Fig. 1F). Furthermore, we assessed the protein expression of ULK1 in EOC cell lines, finding significantly higher expression in human EOC cell lines ES-2, Caov-3, and OVCAR-3 compared to the human normal ovarian epithelial cell line HOSEpiC (Fig. 1G). Collectively, these findings suggest that ULK1 is upregulated in EOC and may serve as a potential prognostic biomarker for EOC patients.

**Table 1:** Relationships between ULK1 expression and clinicopathological parameters in EOC patients.

Parameters	Case number	ULK1 expression		*P* value
		Low (*n* = 39)	High (*n* = 39)	
Age				ns
≤55	40	18	22	
>55	38	21	17	
FIGO stage				***
I/II	23	19	4	
III/IV	55	20	35	
Grade				ns
Well/moderate	15	10	5	
Poor	63	29	34	
Pathological type				ns
Serous	64	31	33	
Clear cell	4	1	3	
Endometrioid	6	4	2	
Mucinous	4	3	1	

*ns* not significant, *FIGO* International Federation of Gynecology and Obstetrics

***P < 0.001.

### ULK1 knockdown inhibits mitophagy, proliferation, and migration of EOC cells in vitro, and growth of xenograft tumors in vivo

**Fig. 2:**
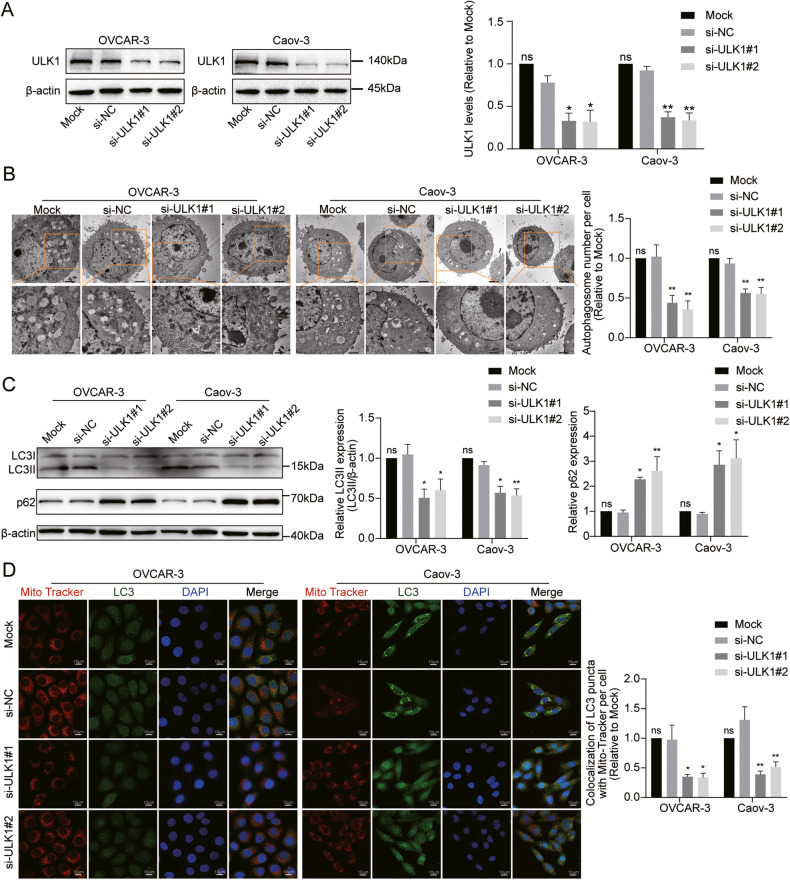
ULK1 knockdown inhibits EOC cells mitophagy in vitro. **A** The efficiency of ULK1 knockdown in OVCAR-3 and Caov-3 cell lines was evaluated through western blot. **B** Representative images of TEM showed the number of autophagosomes in OVCAR-3 and Caov-3 cells after ULK1 knockdown. **C** Protein expression levels of LC3B and p62 in OVCAR-3 and Caov-3 cells after ULK1 knockdown were detected by western blot. **D** Confocal microscopy images of MitoTracker Red staining and LC3 IF staining. ns not significant; *P < 0.05; **P < 0.01; ***P < 0.001.

To investigate the functions of ULK1 in EOC cells, we initially downregulated ULK1 expression using siRNAs in OVCAR-3 and Caov-3 cells (Fig. 2A). TEM revealed fewer autophagosomes in OVCAR-3 and Caov-3 cells following ULK1 knockdown (Fig. 2B). Similarly, western blot assays confirmed that ULK1 knockdown inhibited autophagosome formation (Fig. 2C). To further verify the impact of ULK1 on mitophagy, we analyzed the colocalization of MitoTracker Red and LC3B puncta to assess the co-occurrence of mitochondria with autophagosomes. We observed decreased accumulation of LC3B puncta and reduced colocalization of MitoTracker Red and LC3B puncta after ULK1 downregulation in both cell lines (Fig. 2D). This indicates that ULK1 knockdown inhibits mitophagy in EOC cells.

**Fig. 3:**
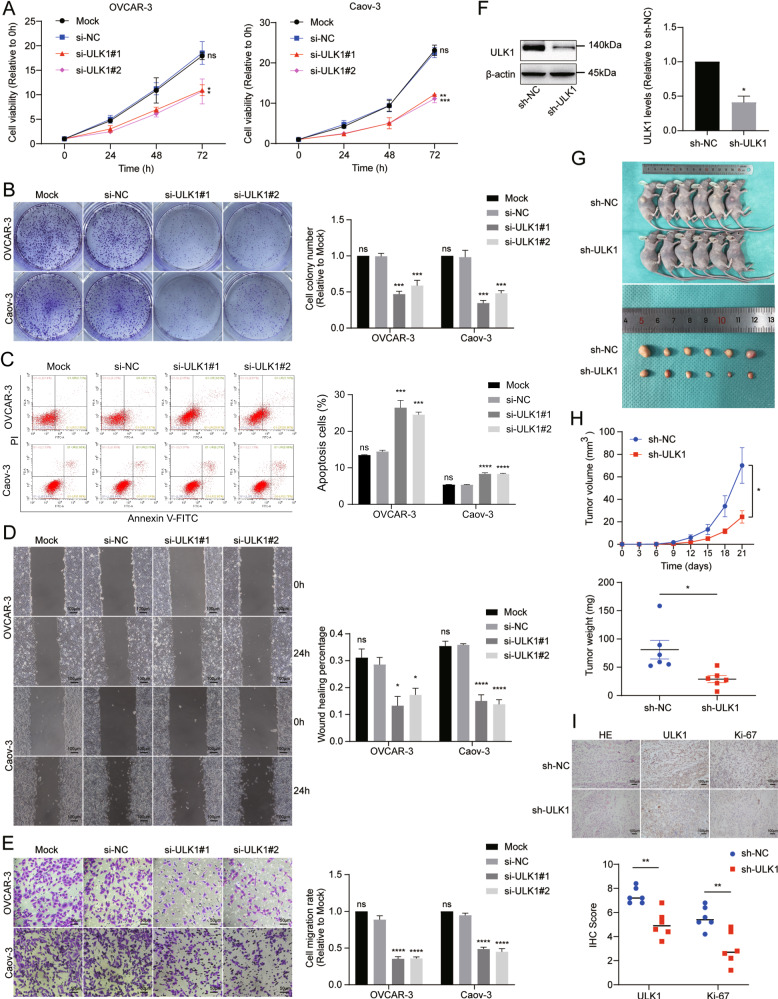
ULK1 knockdown inhibits proliferation and migration of EOC cells in vitro and growth of xenograft tumors in vivo. **A** Cell viability was detected by CCK-8 assays after ULK1 knockdown. **B** Colony formation assays were used to detect the proliferation of OVCAR-3 and Caov-3 cells after ULK1 knockdown. **C** Flow cytometry verified ULK1 functions in OVCAR-3 and Caov-3 cells apoptosis after ULK1 knockdown. **D**, **E** Cell scratch and transwell assays were used to detect cell migration after ULK1 knockdown. **F** Efficiency of ULK1 knockdown in ES-2 cell line was examined by western blot. **G**, **H** The tumor growth curve and the weight of xenograft tumors at the endpoints. **I** Representative images of IHC staining showed the expression of ULK1 and Ki67 of xenograft tumors. ns not significant; *P < 0.05; **P < 0.01; ***P < 0.001; ****P < 0.0001.

To investigate the impact of ULK1 on the malignant biological behavior of EOC cells, we conducted various assays, including CCK-8, colony formation, Transwell, and cell scratch assays, to examine the influence of ULK1 on EOC cells. The CCK-8 and colony formation assays revealed that ULK1 downregulation significantly impeded EOC cell proliferation (Fig. 3A, B). To understand why cell proliferation rates decreased following ULK1 downregulation, we analyzed apoptosis phenotypes in EOC. The annexin V-FITC/PI apoptosis detection assay showed a marked increase in apoptotic cells in the RNA interference groups compared to control groups (Fig. 3C). Additionally, Transwell migration and cell scratch assays indicated that ULK1 downregulation also inhibited the migration of OVCAR-3 and Caov-3 cells (Fig. 3D, E).

To further explore ULK1’s role in EOC growth in vivo, we established xenograft growth models. These models involved subcutaneous injection of ULK1 stable knockdown and control ES-2 cells (Fig. 3F) into female BALB/c nude mice. We observed that the growth of tumors derived from cells with stable ULK1 knockdown was significantly reduced compared to tumors from control cells. Additionally, the stable knockdown of ULK1 also led to a decrease in tumor weight (Fig. 3G, H). IHC also demonstrated decreased expression of the proliferation marker Ki-67 in the sh-ULK1 group (Fig. 3I).

### ULK1 promotes mitophagy, proliferation, and migration of EOC cells in vitro; mitophagy inhibitors can reverse these effects

**Fig. 4:**
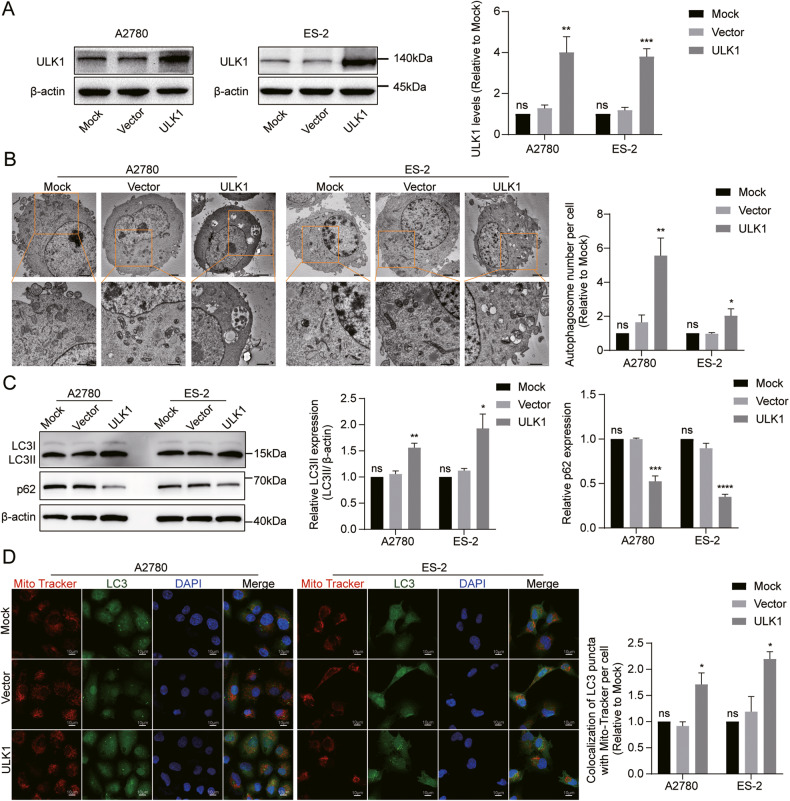
ULK1 overexpression promotes mitophagy of EOC cells in vitro. **A** Efficiency of ULK1 overexpression in A2780 and ES-2 cell lines was evaluated through western blot. **B** Representative images of TEM showed the number of autophagosomes in A2780 and ES-2 cells after ULK1 overexpression. **C** Protein expression levels of LC3B and p62 in A2780 and ES-2 cells after ULK1 overexpression were detected by western blot. **D** Confocal microscopy images of MitoTracker Red staining and LC3 IF staining. ns not significant; *P < 0.05; **P < 0.01; ***P < 0.001.

We established A2780 and ES-2 cell lines with stable ULK1 overexpression through lentiviral infection (Fig. 4A). TEM revealed an increased number of autophagosomes in A2780 and ES-2 cells following ULK1 overexpression (Fig. 4B). Western blot assays indicated that ULK1 upregulation promotes autophagosome formation (Fig. 4C). Consistently, the colocalization of MitoTracker Red and LC3B puncta increased following ULK1 upregulation in both cell lines (Fig. 4D), suggesting that ULK1 upregulation enhances mitophagy in EOC cells.

**Fig. 5:**
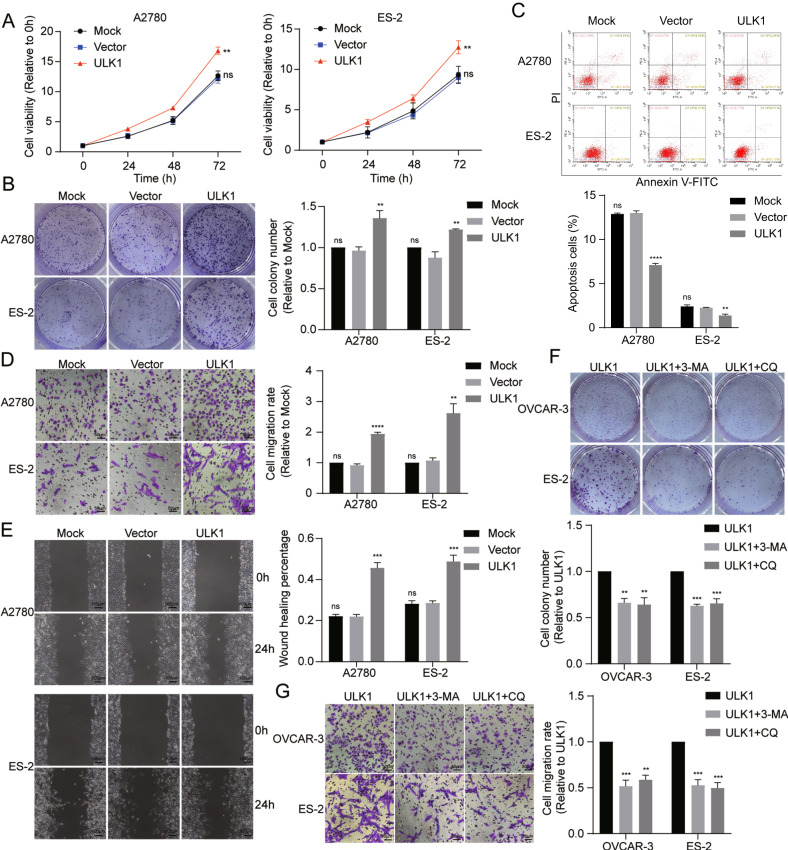
ULK1 overexpression promotes proliferation and migration of EOC cells in vitro; mitophagy inhibitors can reverse these effects. **A** Cell viability was detected by CCK-8 assays after ULK1 overexpression. **B** Colony formation assays were used to detect the proliferation of A2780 and ES-2 cells after ULK1 overexpression. **C** Flow cytometry verified ULK1 functions in A2780 and ES-2 cells apoptosis after ULK1 overexpression. **D**, **E** Transwell and cell scratch assays were used to detect cell migration after ULK1 overexpression. **F**, **G** Colony formation and transwell assays were used to detect the proliferation and migration of OVCAR-3 and ES-2 cells after treated with autophagy inhibitors 3-MA (5 mM) or CQ (30 μM) for 24 h. ns not significant; **P < 0.01; ***P < 0.001; ****P < 0.0001.

CCK-8 and colony formation assays demonstrated that ULK1 overexpression enhances the proliferation ability of EOC cells (Fig. 5A, B). Annexin V-FITC/PI apoptosis detection assays showed a significant decrease in apoptotic cells in the ULK1 overexpressed group compared to control groups (Fig. 5C). Transwell migration and scratch assays indicated significantly increased migration abilities in A2780 and ES-2 cells post-ULK1 overexpression (Fig. 5D, E). These findings collectively suggest that ULK1 may contribute to oncogenic function in EOC by facilitating cellular mitophagy, proliferation, and migration.

To assess whether ULK1-induced mitophagy plays a protective role in EOC cells, we examined cell proliferation and migration abilities in the presence of autophagy inhibitors, 3-Methyladenine (3-MA, 5 mM, M9281, Sigma) or chloroquine (CQ, 30 μM, C6628, Sigma), in ULK1-overexpressing OVCAR-3 and ES-2 cells. Colony formation assays revealed reduced cell proliferation in OVCAR-3 and ES-2 cells overexpressing ULK1 when treated with 3-MA or CQ for 24 h (Fig. 5F). Additionally, these treatments resulted in decreased migration abilities in ULK1-overexpressing OVCAR-3 and ES-2 cells (Fig. 5G). These findings suggest that ULK1 induces protective autophagy and mitophagy, promoting proliferation and migration in EOC cells.

### The stability and expression of ULK1 are enhanced by WTAP-mediated m6A modification

**Fig. 6:**
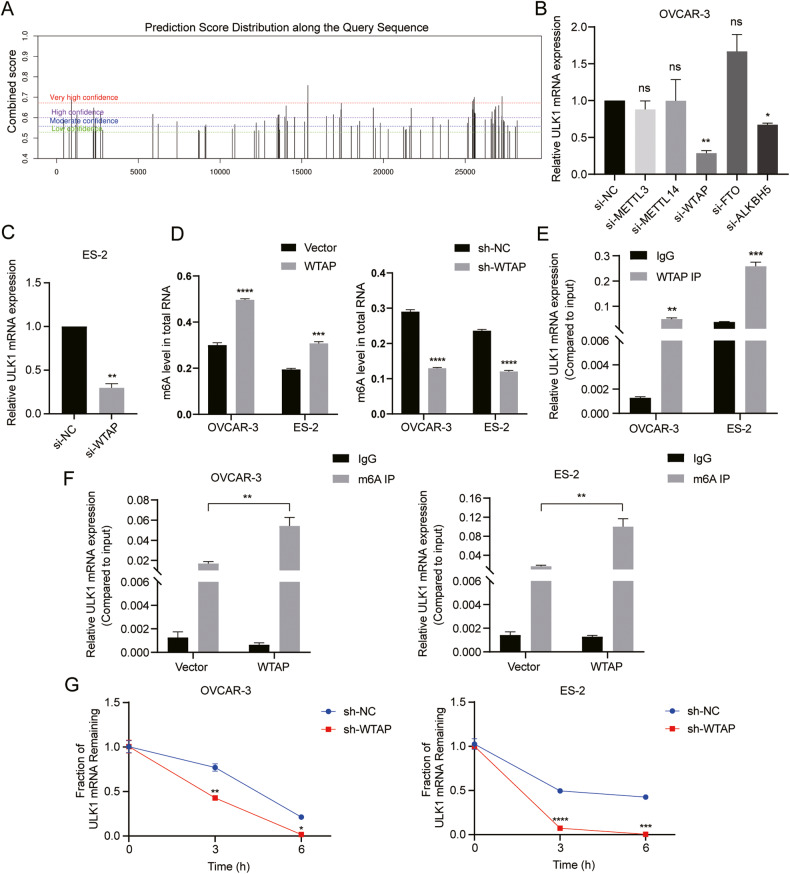
The stability and expression of ULK1 are enhanced by WTAP mediated m6A modification. **A** The predicted m6A modification sites in ULK1 pre-mRNA in the SRAMP database. **B** The ULK1 mRNA expression levels in si-NC, si-METTL3, si-METTL14, si-WTAP, si-FTO, and si-ALKBH5 OVCAR-3 cells were examined by RT-qPCR. **C** The ULK1 mRNA expression levels in si-NC and si-WTAP ES-2 cells were examined by RT-qPCR. **D** m6A levels in total RNA of OVCAR-3 and ES-2 cells after WTAP overexpression and WTAP knockdown were detected by m6A methylation quantification kit. **E** The direct interaction between ULK1 and WTAP was verified by RIP-qPCR assay in OVCAR-3 and ES-2 cells. **F** The enriched m6A modification of ULK1 in WTAP overexpression and control groups was detected by MeRIP-qPCR assay in OVCAR-3 and ES-2 cells. **G** After treatment of OVCAR-3 and ES-2 cells with ACD for 0, 3, and 6 h, ULK1 expression in sh-NC and sh-WTAP groups was detected by RT-qPCR. ns not significant; *P < 0.05; **P < 0.01; ***P < 0.001; ****P < 0.0001.

m6A modification, the most prevalent post-transcriptional modification in eukaryotes, has been implicated in various cancers. Our prior research indicated higher m6A levels in EOC tissues compared to normal ovarian tissues [25]. To investigate the molecular mechanisms underlying ULK1 expression upregulation and malignant phenotype promotion in EOC, we initially utilized the SRAMP website to predict m6A modification sites on ULK1 mRNA. This prediction revealed numerous potential m6A sites on ULK1 (Fig. 6A). Consequently, we employed siRNAs to suppress m6A-regulated enzymes, including WTAP, METTL3, METTL14, ALKBH5, and FTO, in OVCAR-3 cells (Fig. S1A), followed by measuring ULK1 mRNA expression changes using RT-qPCR. The most significant decrease in ULK1 mRNA expression occurred after WTAP knockdown (Fig. 6B), a finding further corroborated in ES-2 cells (Fig. S1B; Fig. 6C).

To ascertain if WTAP regulates ULK1 expression via m6A modification, we assessed m6A levels in ES-2 and OVCAR-3 cells following lentivirus-mediated WTAP overexpression and knockdown. These interventions respectively led to increases and decreases in total RNA m6A levels (Fig. 6D). Additionally, a RIP-qPCR assay confirmed a direct interaction between ULK1 and WTAP (Fig. 6E), aligning with the predicted protein-RNA binding between WTAP and ULK1 from the RM2Target database (Fig. S1C). We then examined the m6A levels of ULK1 in OVCAR-3 and ES-2 cells with WTAP overexpression using a MeRIP-qPCR assay. The results demonstrated that WTAP upregulation augmented ULK1’s m6A modifications in both cell lines compared to controls (Fig. 6F). An actinomycin D experiment was subsequently conducted to assess if WTAP influences ULK1 mRNA stability. The findings revealed reduced ULK1 mRNA stability in WTAP-downregulated OVCAR-3 and ES-2 cells (Fig. 6G). These results collectively suggest that WTAP stabilizes and positively regulates ULK1 expression by enhancing its m6A modification.

### WTAP exerts oncogenic effects through ULK1-mediated mitophagy

**Fig. 7:**
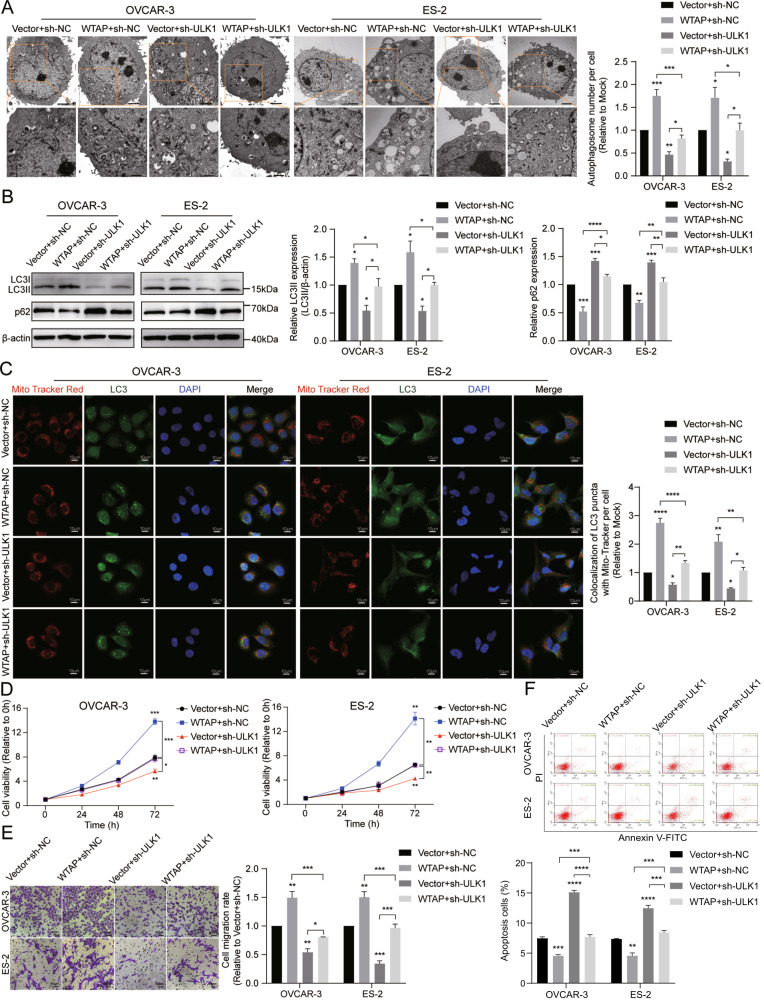
WTAP exerts oncogenic effects through ULK1-mediated mitophagy. **A** Representative images of TEM showed the number of autophagosomes in OVCAR-3 and ES-2 cells. **B** Protein expression levels of LC3B and p62 in OVCAR-3 and ES-2 cells were detected by western blot assay. **C** Representative confocal microscopy images of MitoTracker Red staining and LC3 IF staining. **D** Cell viability was detected by CCK-8 assays. **E** Transwell assay was used to detect cell migration. **F** Flow cytometry was used to detect cell apoptosis. *P < 0.05; **P < 0.01; ***P < 0.001; ****P < 0.0001.

To determine if WTAP is linked to ULK1-induced proliferation and migration in EOC cells, we performed rescue experiments. Figure 7A–C demonstrate that downregulation of ULK1 expression partially counteracted the enhancement of autophagy and mitophagy induced by WTAP overexpression, as evidenced by TEM, colocalization of MitoTracker Red and LC3B puncta, and western blot assays. Further, CCK-8 and Transwell assays indicated that the increased proliferation and migration abilities of EOC cells, spurred by WTAP overexpression, were partially reversed by ULK1 downregulation (Fig. 7D, E). Additionally, the annexin V-FITC/PI apoptosis detection assay showed that the suppression of apoptosis induced by WTAP overexpression was partially negated by ULK1 downregulation (Fig. 7F). Overall, the outcomes of these rescue experiments suggest that WTAP acts upstream of ULK1 in EOC cells. Reducing ULK1 expression and its mediated mitophagy can mitigate the tumor-promoting effect of WTAP overexpression.

### WTAP enhances the stability of ULK1 mRNA in an IGF2BP3-dependent manner

**Fig. 8:**
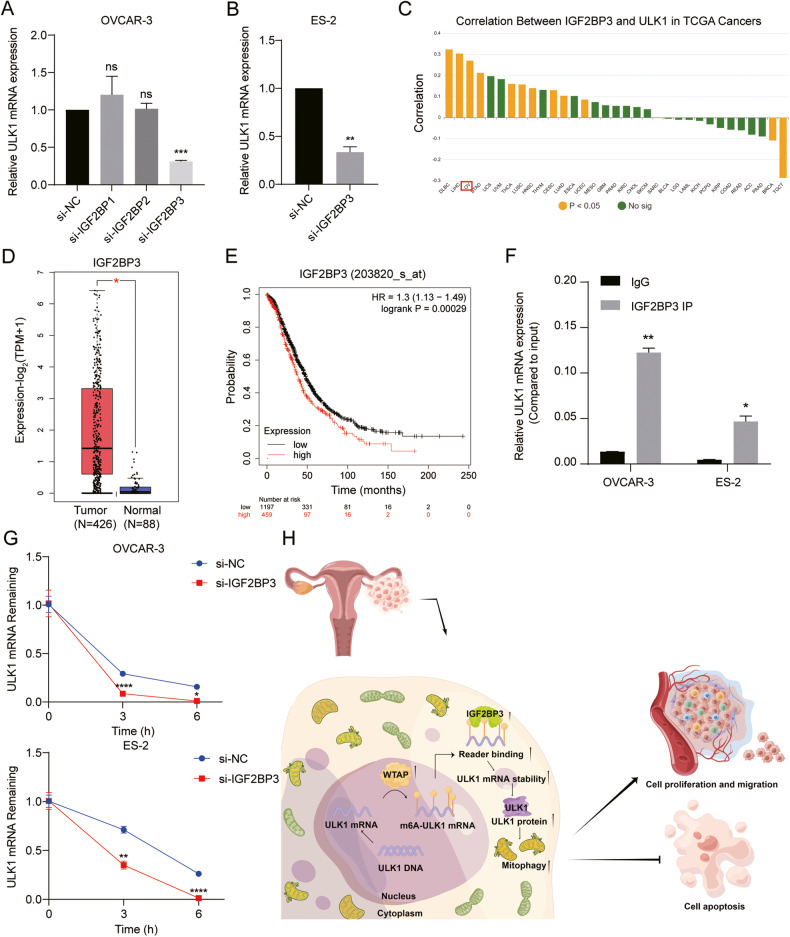
WTAP enhances the stability of ULK1 mRNA in an IGF2BP3 dependent manner. **A** The ULK1 mRNA expression levels in si-NC, si-IGF2BP1, si-IGF2BP2, and si-IGF2BP3 OVCAR-3 cells were examined by RT-qPCR. **B** The ULK1 mRNA expression levels in si-NC and si-IGF2BP3 ES-2 cells were examined by RT-qPCR. **C** RM2Target database predicted a positive correlation between IGF2BP3 and ULK1 expression in TCGA ovarian cancer. **D** The mRNA expression levels of IGF2BP3 in OV and normal ovarian tissues from the GEPIA database. **E** Kaplan–Meier plots for the OS of OV patients according to IGF2BP3 expression. **F** The direct interaction between ULK1 and IGF2BP3 was verified by RIP-qPCR assay in OVCAR-3 and ES-2 cells. **G** After treatment of OVCAR-3 and ES-2 cells with ACD for 0, 3, and 6 h, ULK1 expression in si-NC and si-IGF2BP3 groups was detected by RT-qPCR. **H** Schematic diagram of the present study (by Figdraw). ns not significant; *P < 0.05; **P < 0.01; ***P < 0.001; ****P < 0.0001.

m6A-modified RNA is recognized by “readers” to perform its biological functions. The IGF2BPs family, one of these “reader” protein families, binds to m6A-modified mRNA, influencing mRNA stability [31]. To investigate this, we used siRNAs to knock down IGF2BP1/2/3 in OVCAR-3 cells (Fig. S2A) and observed changes in ULK1 mRNA expression. A significant decrease in ULK1 expression was noted following the knockdown of IGF2BP3 (Fig. 8A). This regulatory effect of IGF2BP3 on ULK1 was further confirmed in ES-2 cells (Fig. S2B; Fig. 8B). Correspondingly, the RM2Target database predicts a positive correlation between IGF2BP3 and ULK1 expression in the TCGA-OV dataset (Fig. 8C), and IGF2BP3 has been shown to bind to and regulate ULK1 at the protein-RNA level (Fig. S2C). Additionally, the GEPIA database indicates significantly higher mRNA expression of IGF2BP3 in OV tissues compared to normal ovarian tissues (Fig. 8D). Furthermore, the Kaplan–Meier plotter database reveals a negative correlation between IGF2BP3 expression and overall survival (OS) in OV patients (Fig. 8E). These findings lead us to speculate that IGF2BP3 is the primary reader in the IGF2BPs family that influences ULK1 stability. This was further validated using RIP-qPCR assays, confirming the direct interaction between IGF2BP3 and ULK1 in both OVCAR-3 and ES-2 cells (Fig. 8F). The actinomycin D experiment demonstrated that ULK1 mRNA stability decreases following IGF2BP3 knockdown (Fig. 8G). In summary, these results suggest that WTAP enhances the m6A modification of ULK1 mRNA through methylation, promoting IGF2BP3’s recognition and binding, which in turn increases the stability of ULK1 mRNA and augments ULK1 expression in EOC (Fig. 8H).

## Discussion

OV has the poorest prognosis among the three common gynecological malignancies, exhibiting a 5-year survival rate of less than 50% [32]. EOC, constituting over 90% of all diagnosed OV cases, is the predominant subtype of this malignancy [33]. Most EOC patients are diagnosed at an advanced stage, with a high likelihood of recurrence within a few years after debulking surgery and platinum-taxane chemotherapy. This recurrence is especially prevalent in advanced stages and significantly affects patient survival. Although recent developments in poly (ADP-ribose) polymerase inhibitors and bevacizumab have been effective in prolonging PFS, they have not been successful in extending OS [34, 35]. This highlights the necessity for further investigation into the mechanisms driving EOC progression and the development of more effective treatments.

ULK1 is a key protein kinase in autophagy regulation. It initiates autophagy by forming a stable ULK1-ATG13-FIP200-ATG101 complex [13]. Various studies have detailed the structural characteristics and biological functions of ULK1, as well as its regulatory pathways in autophagy and its association with different diseases, including cancer. Notably, a range of small molecule compounds targeting ULK1 have shown promising therapeutic effects on these diseases [13]. Apart from its significant role in autophagy, ULK1 has also been identified as a specific regulator of mitophagy [15]. Similar to autophagy, mitophagy plays a complex, dual role in cancer, but its specific impact on EOC remains largely unexplored.

Our previous bioinformatics analysis of the TCGA-OV dataset revealed mutations and CNV in mitophagy-related genes. Notably, some of these genes are associated with the prognosis of OV patients, with ULK1 being identified as a prognostic risk factor [12]. Similarly, Singha B et al. demonstrated a significant negative correlation between ULK1 mRNA expression and patient survival in stage III and IV serous OV patients, using data from the Gene Expression Omnibus (GEO) and TCGA databases [36]. They also provided substantial evidence that spheroids of high-grade serous ovarian cancer (HGSOC) exhibit increased ULK1 protein expression, correlating with the initiation of autophagy. This autophagy activation contributes to the survival of spheroid cells and overall disease progression [36].

Therefore, we validated ULK1 expression in EOC. Our results showed significantly higher ULK1 expression in EOC tissues compared to normal ovarian tissues, with its high expression correlating with advanced FIGO staging. Similarly, ULK1 expression was significantly elevated in EOC cell lines compared to normal ovarian epithelial cell lines. Furthermore, we explored the function of ULK1 in EOC cells using gain-of-function and loss-of-function approaches. Our findings revealed that ULK1 overexpression stimulates cell proliferation and migration by inducing protective mitophagy in EOC cells. In contrast, downregulating ULK1 expression produced opposite effects, further validated by in vivo experiments in a xenograft mouse model. These results affirm that ULK1 and its mediated mitophagy play a cancer-promoting role in EOC and may represent potential therapeutic targets for EOC patients.

Previous studies have demonstrated that ULK1 regulation occurs at the transcriptional, post-transcriptional, and post-translational levels, impacting its expression, activity, and function [13]. However, the specific mechanism governing ULK1 expression in EOC and its subsequent pro-tumor effect remains unclear. m6A methylation modification, the most common post-transcriptional modification in eukaryotic mRNA, is a dynamic and reversible process controlled by “writers,” “erasers,” and “readers.” Alterations in the m6A modification of target genes can influence their expression and functionality by affecting mRNA stability, translation, and degradation processes [18]. Numerous studies have identified that abnormal m6A modification plays a role in the onset and progression of cancer. Our prior research indicated elevated m6A modification levels in EOC tissue [25], and predictions from the SRAMP website identified numerous m6A modification sites on ULK1 mRNA. Based on these findings, we hypothesize that m6A modification may play a role in regulating ULK1 expression in EOC.

To further investigate the m6A-regulated enzymes that influence ULK1 regulation, we conducted knockdown experiments on the methyltransferases METTL3, METTL14, and WTAP, as well as the demethylases ALKBH5 and FTO. We observed that ULK1 expression decreased most notably following the knockdown of WTAP. Additionally, through RIP and MERIP experiments, we established a direct interaction between WTAP and ULK1, clarifying WTAP’s role in modulating the m6A modification level of ULK1 mRNA. Prior studies have indicated that WTAP can regulate the mRNA stability of its target genes [37–41]. In line with these findings, our study also showed that WTAP affects the stability of ULK1 mRNA. Hence, we suggest that WTAP regulates ULK1 expression by enhancing ULK1 mRNA stability in an m6A-dependent manner in EOC.

m6A modification typically necessitates recognition by specific readers to manifest its effects. Previous research has identified IGF2BPs as readers that regulate mRNA stability [31, 42–44]. In our study, we employed siRNAs to knock down the expression of IGF2BP1/2/3 and observed a significant decrease in ULK1 expression following the knockdown of IGF2BP3. Additionally, bioinformatics analysis of the TCGA-OV dataset unveiled a significant positive correlation between the expressions of IGF2BP3 and ULK1. Consistently, analysis from the GEPIA database showed a significant increase in IGF2BP3 expression in OV, with its elevated levels associated with reduced OS time in patients. We further confirmed a direct interaction between IGF2BP3 and ULK1 using RIP experiments. The role of IGF2BP3 in stabilizing ULK1 mRNA was verified through actinomycin D experiments.

In addition, previous studies have revealed that the effects of METTL3 and METTL14, key components of the m6A MTC, are related to *TP53* status and p53 signaling pathway [45–48]. As the gene with the highest mutation frequency in OV, especially in HGSOC [49, 50], *TP53* has been studied extensively. It is a tumor suppressor gene, and its mutation may result in multiple potential functional outcomes: loss of wild-type function, gain of oncogenic function, dominant negative, and no effect [49]. In the present study, a collective of four EOC cell lines were employed, wherein three of them exhibited distinct *TP53* mutations, except for A2780 [51]. No differential expression of WTAP and ULK1 was found in the four EOC cell lines. Meanwhile, the bioinformatics analysis conducted did not yield evidence supporting an interaction between the WTAP/IGF2BP3/ULK1 axis and TP53 in EOC (Fig. S3). This could potentially be attributed to different methyltransferases or different cancer types, thus warranting further investigation in future studies.

Our research has some limitations. Firstly, although abundant m6A modification sites were predicted on ULK1 mRNA through the SRAMP website, we did not validate the specific binding sites of WTAP or IGF2BP3 to ULK1. Secondly, we did not further investigate the specific signaling pathways through which the WTAP/IGF2BP3/ULK1 axis regulates mitophagy in EOC cells, and these issues require further exploration.

In summary, our findings indicate that ULK1 is upregulated in EOC, and its high expression correlates with poor survival outcomes in EOC patients. Functionally, ULK1 facilitates mitophagy and the progression of EOC. Mechanistically, WTAP collaborates with IGF2BP3 to enhance the expression of the target gene ULK1 by increasing its mRNA stability in an m6A-dependent manner. Crucially, our study suggests that targeting the WTAP/IGF2BP3/ULK1 axis presents a viable therapeutic approach for EOC.

### Supplementary information

Table S1

Table S2

Table S3

Figure S1

Figure S2

Figure S3

Supplementary figure legends

Original Data File

The information of patient samples

reproducibility checklist

## Data Availability

The datasets used and/or analyzed during the current study are available from the corresponding author on reasonable request.
