# Supplementary material for: Regulation of ULK1 by WTAP/IGF2BP3 axis enhances mitophagy and progression in epithelial ovarian cancer
Source: Cell Death Dis. 2024 Jan 29;15(1):97. doi: 10.1038/s41419-024-06477-0 (PMC10824720; doi:10.1038/s41419-024-06477-0)
Supplement: Supplementary file 1 — Table S1 [file 41419_2024_6477_MOESM1_ESM.docx]

| **Reagent or resource** | **Source** | **Identifier** |
| --- | --- | --- |
| Rabbit Polyclonal Antibody for ULK1 (IHC, WB) | Proteintech | Cat No : 20986-1-AP |
| Mouse Monoclonal Antibody for β-actin (WB) | ZSGB-BIO | Cat No : TA-09 |
| Rabbit Monoclonal Antibody for LC3B (WB) | Cell Signaling Technology | Cat No : 3868S |
| Rabbit Polyclonal Antibody for p62 (WB) | Proteintech | Cat No : 18420-1-AP |
| Mouse Monoclonal Antibody for p53 (WB) | Proteintech | Cat No : 60283-2-Ig |
| Mouse Monoclonal Antibody for WTAP (WB) | Proteintech | Cat No : 60188-1-Ig |
