# Supplementary material for: Regulation of ULK1 by WTAP/IGF2BP3 axis enhances mitophagy and progression in epithelial ovarian cancer
Source: Cell Death Dis. 2024 Jan 29;15(1):97. doi: 10.1038/s41419-024-06477-0 (PMC10824720; doi:10.1038/s41419-024-06477-0)
Supplement: Supplementary file 2 — Table S2 [file 41419_2024_6477_MOESM2_ESM.docx]

|  | Forward (5' to 3') | Reverse (5' to 3') |
| --- | --- | --- |
| β-actin | CCTGGCACCCAGCACAAT | GGGCCGGACTCGTCATAC |
| ULK1 | GGCAAGTTCGAGTTCTCCCG | TAATGCACTTGACGGCGACC |
| METTL3 | GTCAGGGCTGGGAGACTAGGATG | CAATGCTGCCTCTGGATTCCGTAG |
| METTL14 | ACCAAAATCGCCTCCTCCCAAATC | AGCCACCTCTTTCTCCTCGGAAG |
| WTAP | AGGGCAACACAACCGAAGATGAC | ACCACTACCTCCTCTGCCAGTTC |
| FTO | GTTCACAACCTCGGTTTAGTTC | CATCATCATTGTCCACATCGTC |
| ALKBH5 | TCCTTTCCCTTCCCTTCTCCACTG | TGAAGCGGAGGAGGCACCAG |
| IGF2BP1 | GCGGCCAGTTCTTGGTCAA | TTGGGCACCGAATGTTCAATC |
| IGF2BP2 | AGTGGAATTGCATGGGAAAATCA | CAACGGCGGTTTCTGTGTC |
| IGF2BP3 | TATATCGGAAACCTCAGCGAGA | GGACCGAGTGCTCAACTTCT |
