# Supplementary material for: Regulation of ULK1 by WTAP/IGF2BP3 axis enhances mitophagy and progression in epithelial ovarian cancer
Source: Cell Death Dis. 2024 Jan 29;15(1):97. doi: 10.1038/s41419-024-06477-0 (PMC10824720; doi:10.1038/s41419-024-06477-0)
Supplement: Supplementary file 3 — Table S3 [file 41419_2024_6477_MOESM3_ESM.docx]

|  | sense（5'-3'） | antisense（5'-3'） |
| --- | --- | --- |
| si-ULK1#1 | GGCUGAAUGAGCUGUACAATT | UUGUACAGCUCAUUCAGCCTT |
| si-ULK1#2 | UACACGCCAUCUCCUCAAGUUTT | AACUUGAGGAGAUGGCGUGUATT |
| si-METTL3 | GGUUGGUGUCAAAGGAAAUTT | AUUUCCUUUGACACCAACCTT |
| si-METTL14 | GGACUUGGGAUGAUAUUAUTT | AUAAUAUCAUCCCAAGUCCTT |
| si-WTAP | GCGAAGUGUCGAAUGCUUATT | UAAGCAUUCGACACUUCGCTT |
| si-ALKBH5 | GCUUCAGCUCUGAGAACUATT | UAGUUCUCAGAGCUGAAGCTT |
| si-FTO | GGCAAUCGAUACAGAAAGUTT | ACUUUCUGUAUCGAUUGCCTT |
| si-IGF2BP1 | GCUCCCUAUAGCUCCUUUATT | UAAAGGAGCUAUAGGGAGCTT |
| si-IGF2BP2 | CAGUUUGAGAACUACUCCUTT | AGGAGUAGUUCUCAAACUGTT |
| si-IGF2BP3 | GCUGCUGAGAAGUCGAUUATT | UAAUCGACUUCUCAGCAGCTT |
