# Supplementary figures and images for: Regulation of ULK1 by WTAP/IGF2BP3 axis enhances mitophagy and progression in epithelial ovarian cancer
Source: Cell Death Dis. 2024 Jan 29;15(1):97. doi: 10.1038/s41419-024-06477-0 (PMC10824720; doi:10.1038/s41419-024-06477-0)

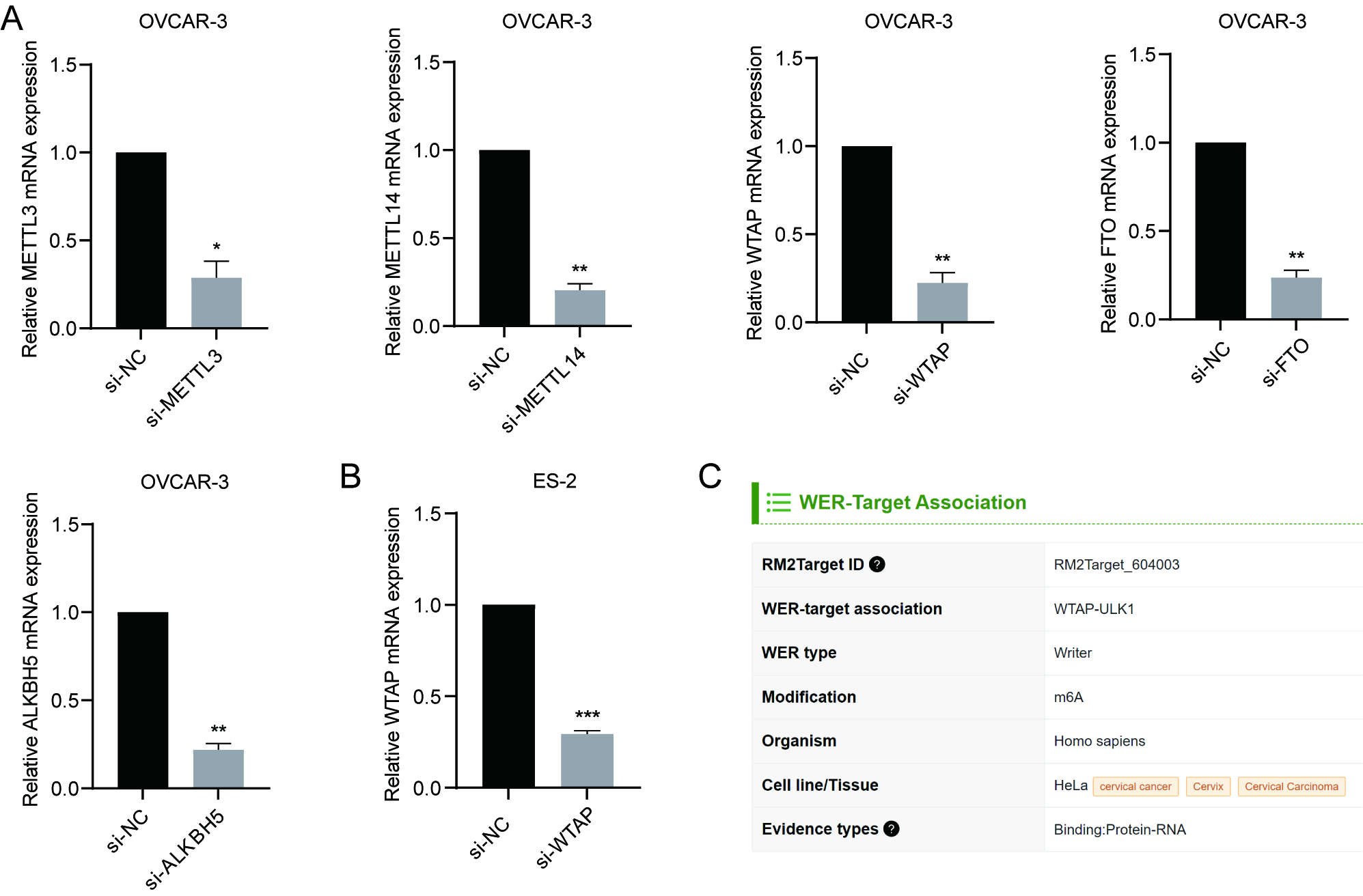

Supplement: Supplementary file 4 — Figure S1 [file 41419_2024_6477_MOESM4_ESM.tif]

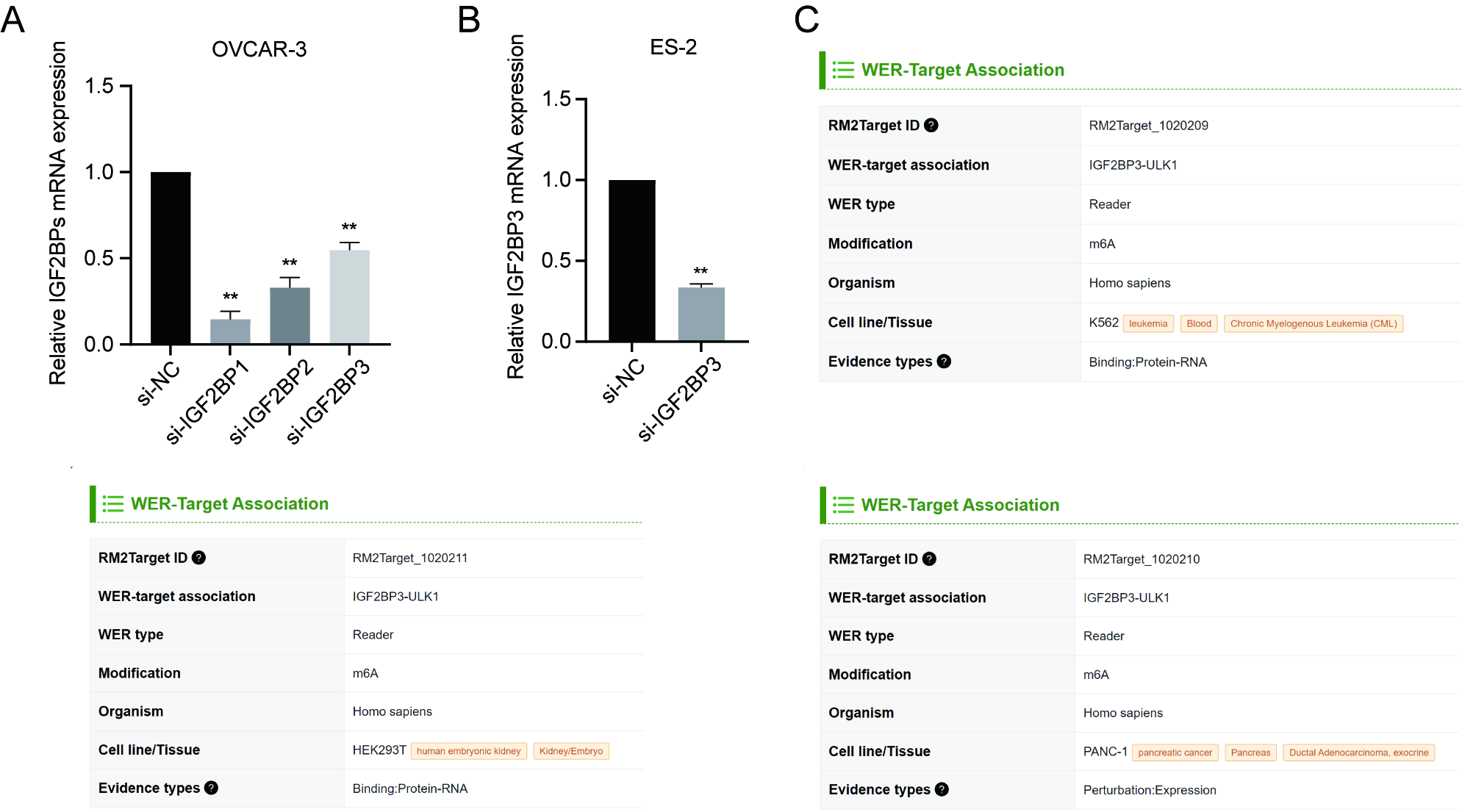

Supplement: Supplementary file 5 — Figure S2 [file 41419_2024_6477_MOESM5_ESM.tif]

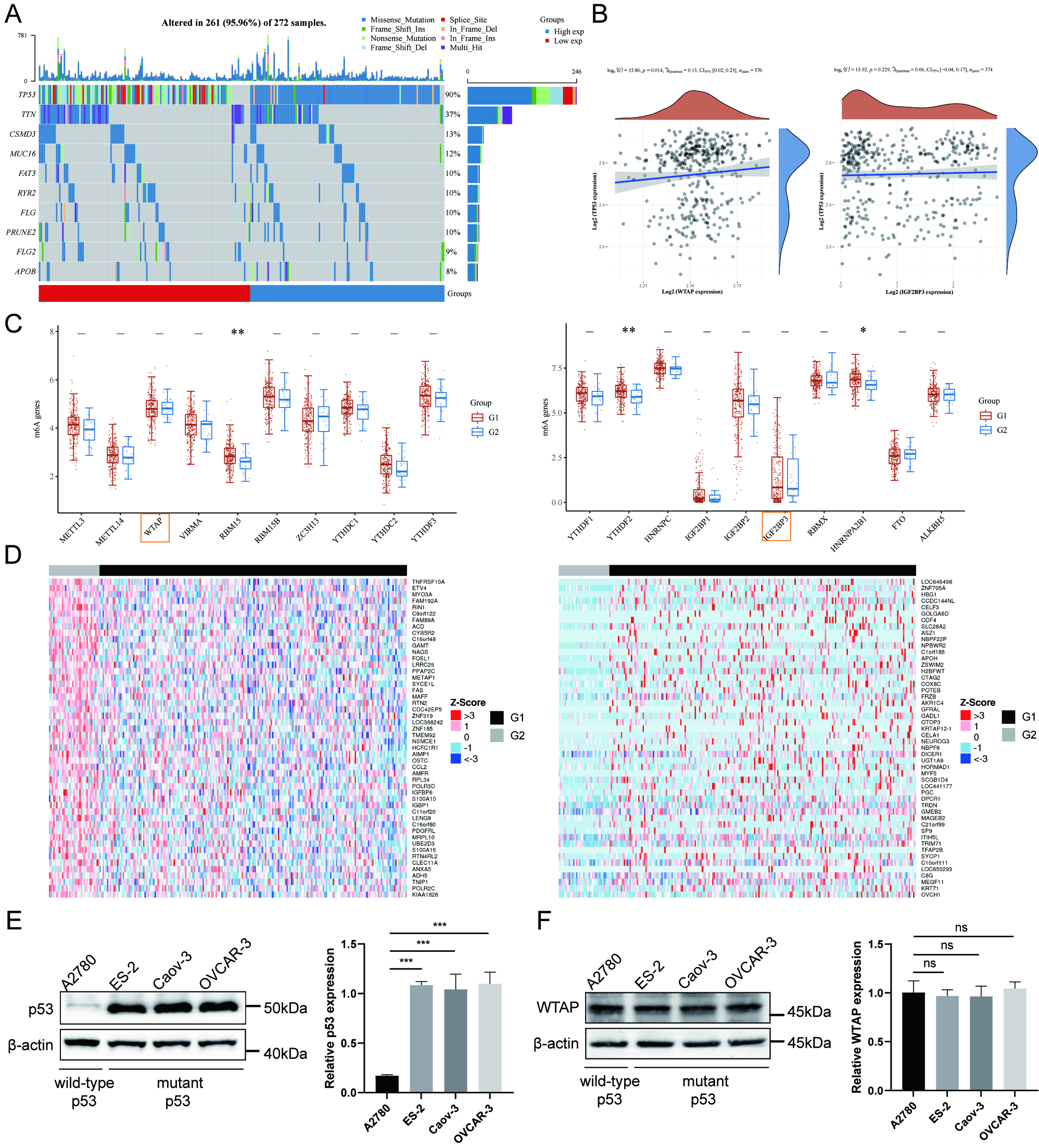

Supplement: Supplementary file 6 — Figure S3 [file 41419_2024_6477_MOESM6_ESM.tif]
