# Supplementary material for: Regulation of ULK1 by WTAP/IGF2BP3 axis enhances mitophagy and progression in epithelial ovarian cancer
Source: Cell Death Dis. 2024 Jan 29;15(1):97. doi: 10.1038/s41419-024-06477-0 (PMC10824720; doi:10.1038/s41419-024-06477-0)
Supplement: Supplementary file 7 — Supplementary figure legends [file 41419_2024_6477_MOESM7_ESM.docx]

**Supplementary figure legends**

**Figure S1.** **(A, B)** Efficiency of m6A-regulated enzymes knockdown in OVCAR-3 and ES-2 cell lines was examined by RT-qPCR. **(C)** The predicted protein-RNA binding between WTAP and ULK1 in the RM2Target database.

**Figure S2.** **(A, B)** Efficiency of IGF2BPs knockdown in OVCAR-3 and ES-2 cell lines was examined by RT-qPCR. **(C)** The predicted protein-RNA binding and regulatory relationships between IGF2BP3 and ULK1 in the RM2Target database.

**Figure S3.** **(A)** Oncoplot showed the somatic landscape of TCGA-OV cohort. Top10 genes were ordered by their mutation frequencies. **(B)** The expression correlation between WTAP and TP53, as well as IGF2BP3 and TP53, was analysed with Spearman. **(C)** The expression distribution of m6A-related genes in *TP53* mutant (Group1) and *TP53* wild-type (Group2) OV. **(D)** The heatmap showed the top 50 genes negatively and positively correlated to the *TP53* mutation in TCGA-OV dataset. **(E, F)** The expression levels of p53 and WTAP in human EOC cell lines A2780, ES-2, Caov-3, and OVCAR-3 were detected by western blot. ns, not significant; * *P* < 0.05; ** *P* < 0.01; *** *P* < 0.001.
