# Supplementary material for: Regulation of ULK1 by WTAP/IGF2BP3 axis enhances mitophagy and progression in epithelial ovarian cancer
Source: Cell Death Dis. 2024 Jan 29;15(1):97. doi: 10.1038/s41419-024-06477-0 (PMC10824720; doi:10.1038/s41419-024-06477-0)

Figure 1A

ULK1

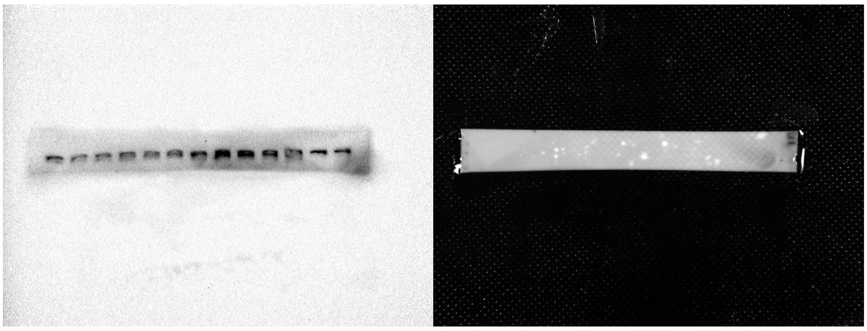

β-actin

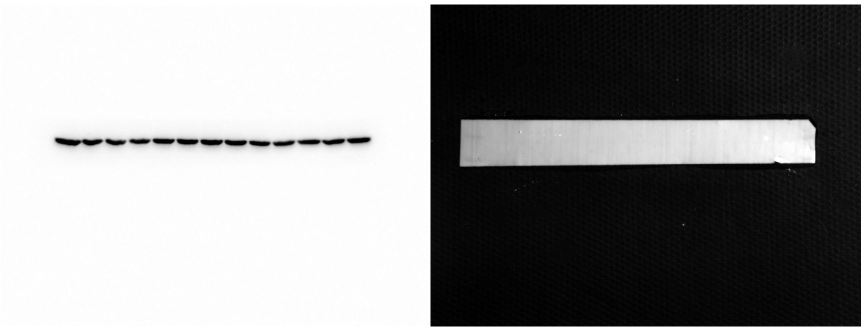

ULK1

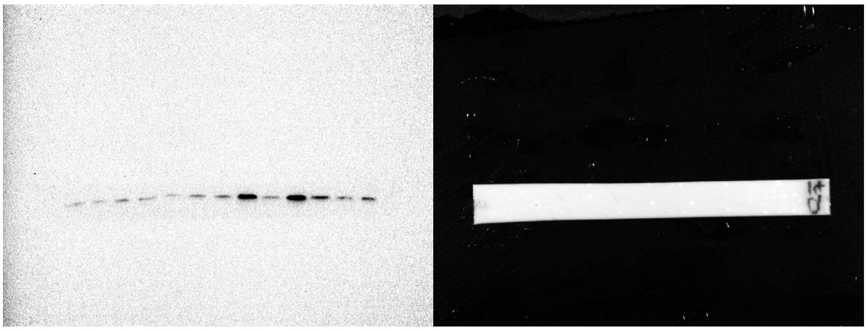

β-actin

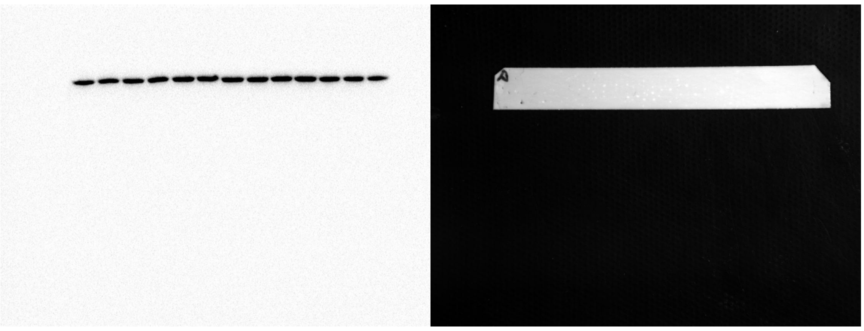

ULK1

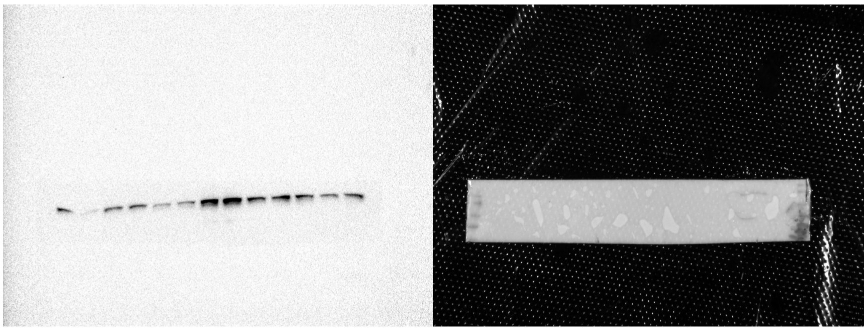

β-actin

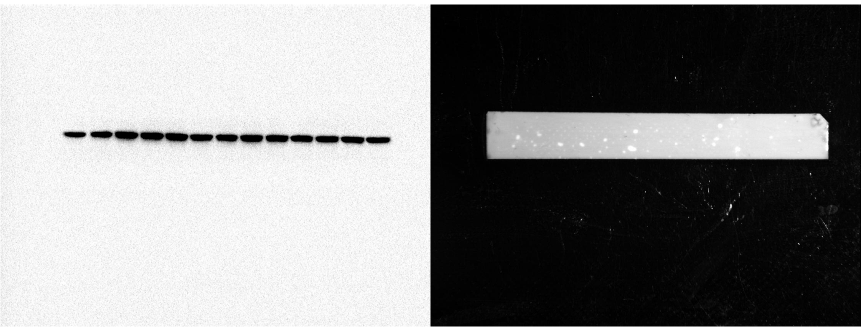

ULK1

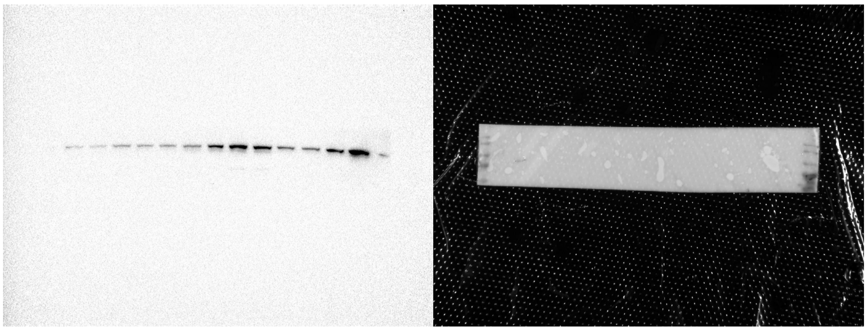

β-actin

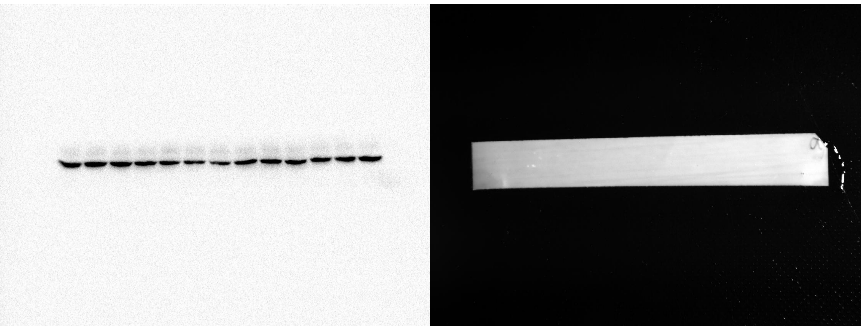

ULK1

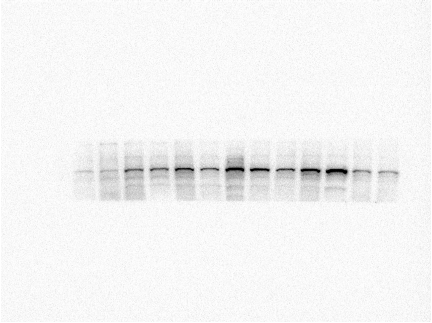

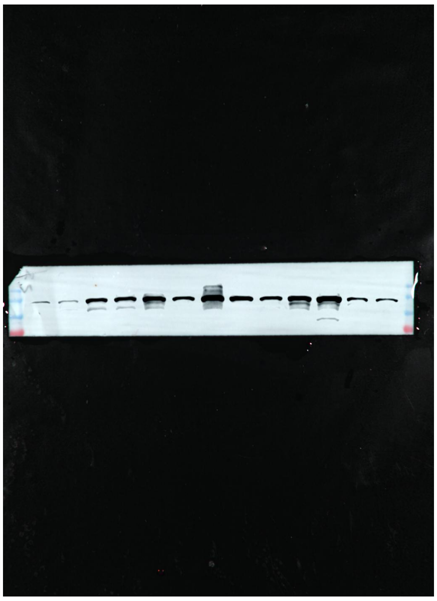

β-actin

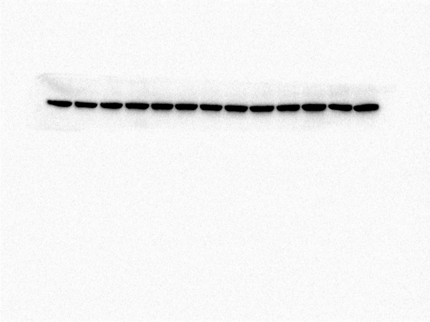

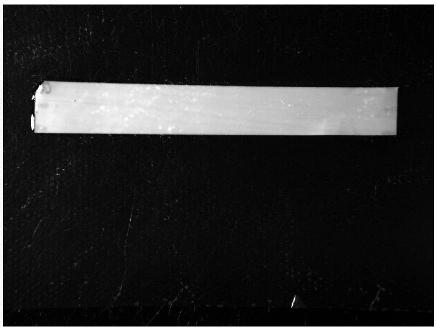

Figure 1G

ULK1

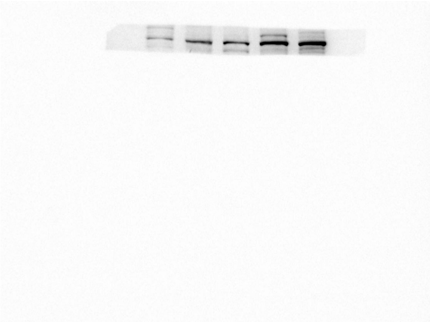

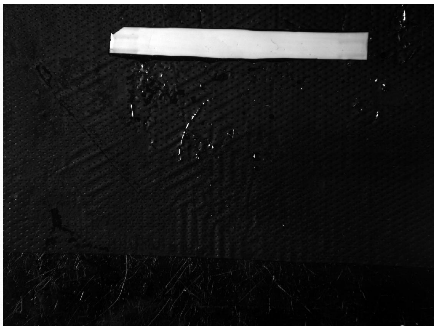

β-actin

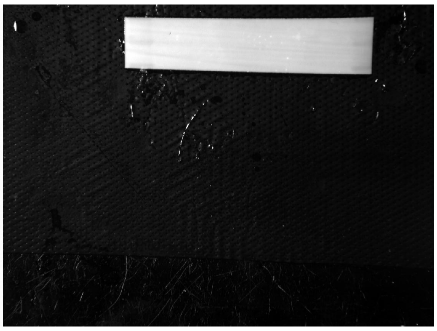

ULK1+β-actin

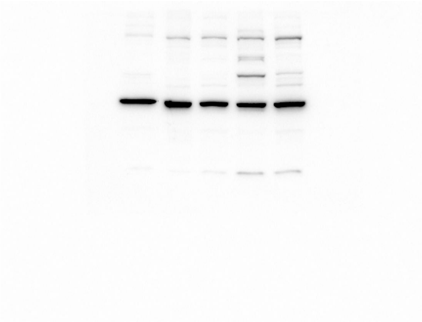
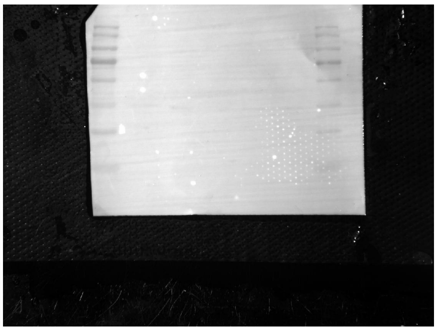

ULK1+β-actin

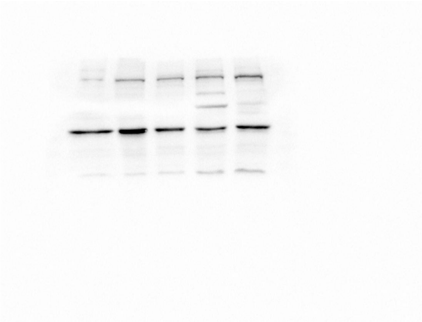
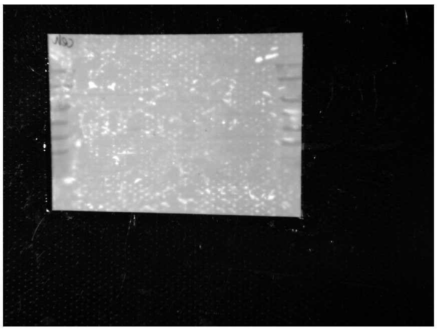

Figure 2A

OVCAR-3

ULK1

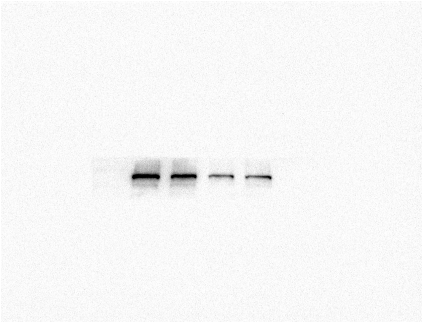
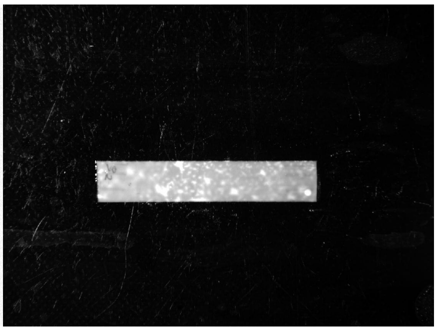

OVCAR-3

β-actin

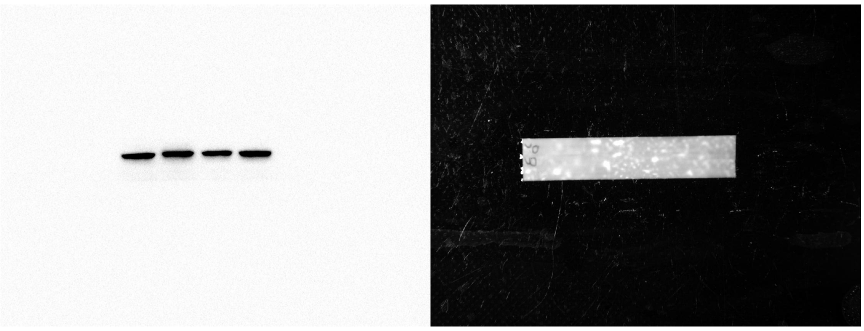

Caov-3
ULK1

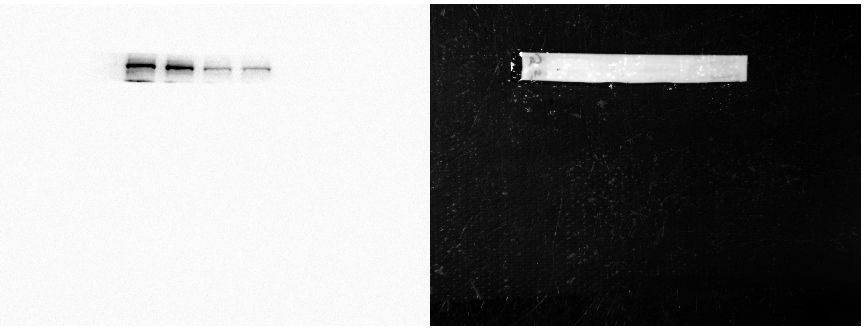

Caov-3
β-actin

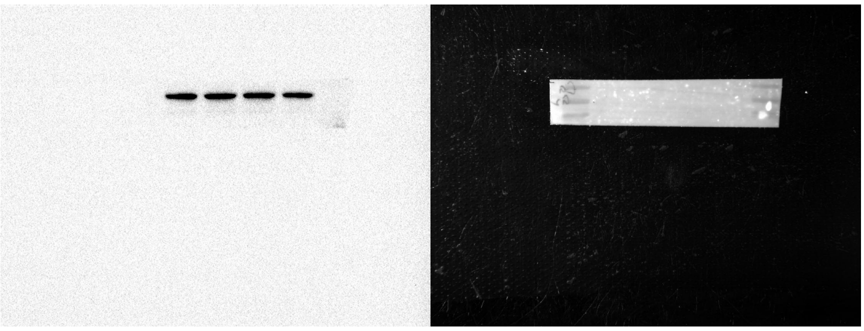

Figure 2C
LC3

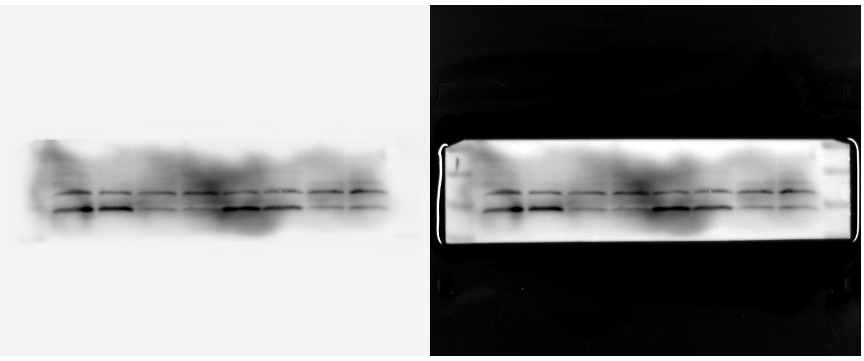

p62

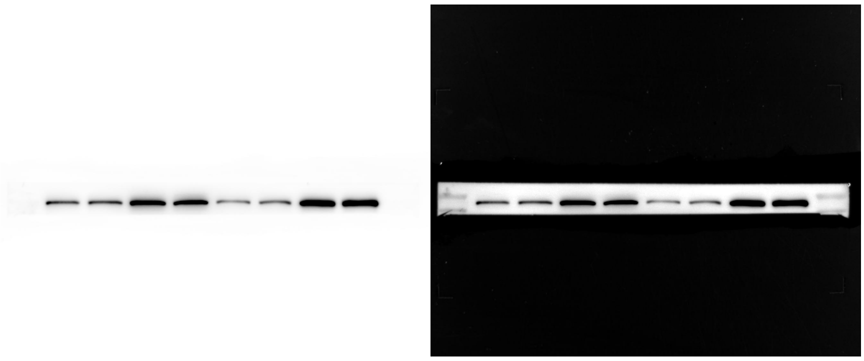

β-actin

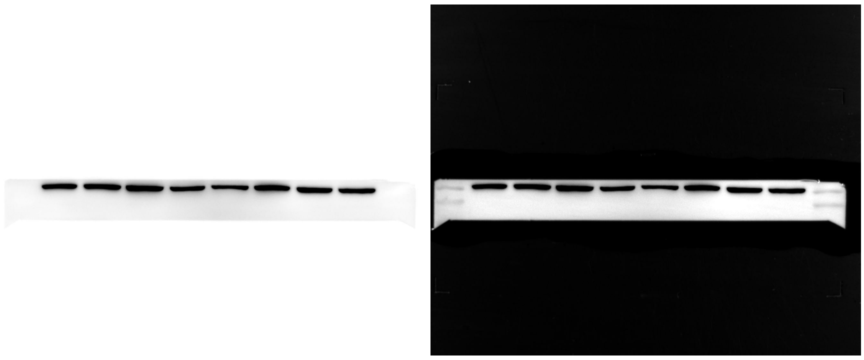

Figure 3F

ES-2

ULK1

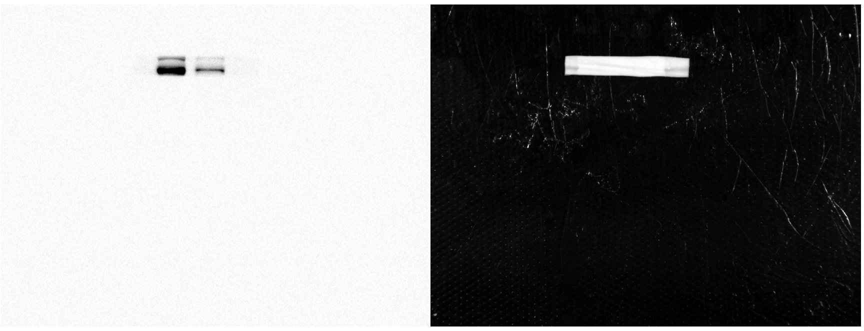

ES-2

β-actin

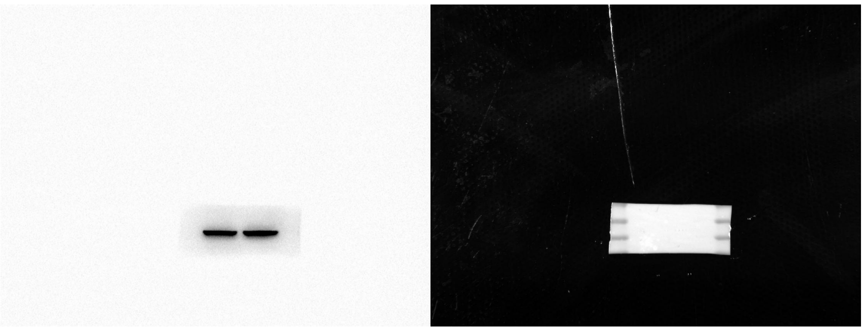

Figure 4A
A2780
ULK1

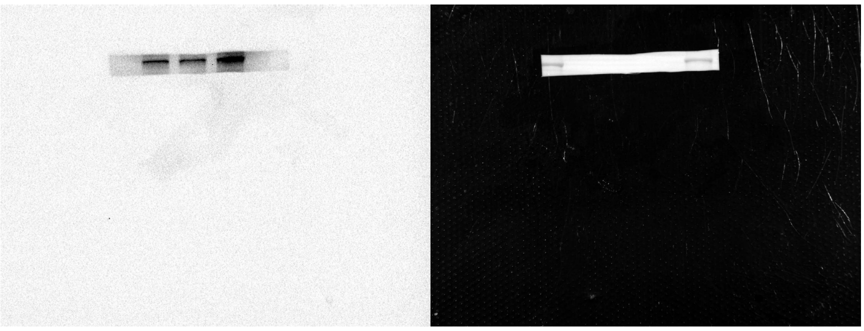

A2780
β-actin

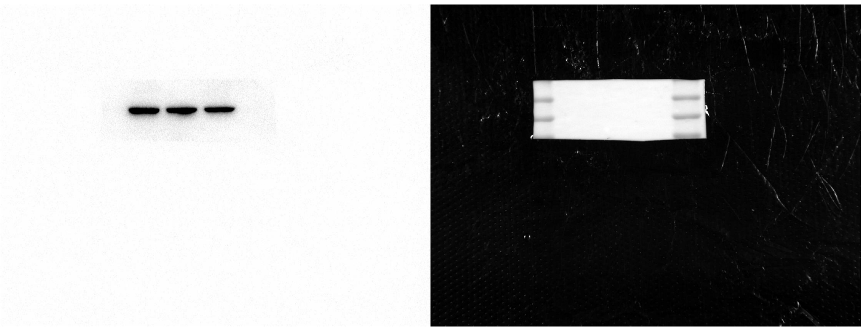

ES-2
ULK1

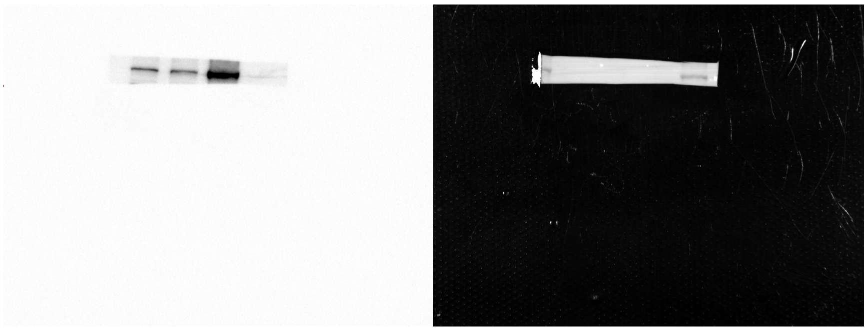

ES-2
β-actin

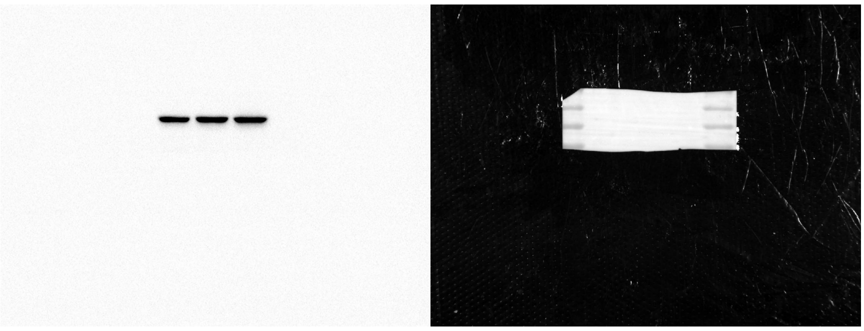

Figure 4C

LC3

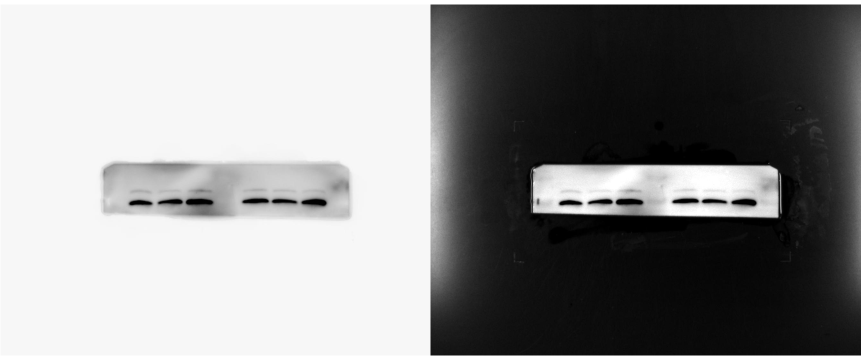

p62

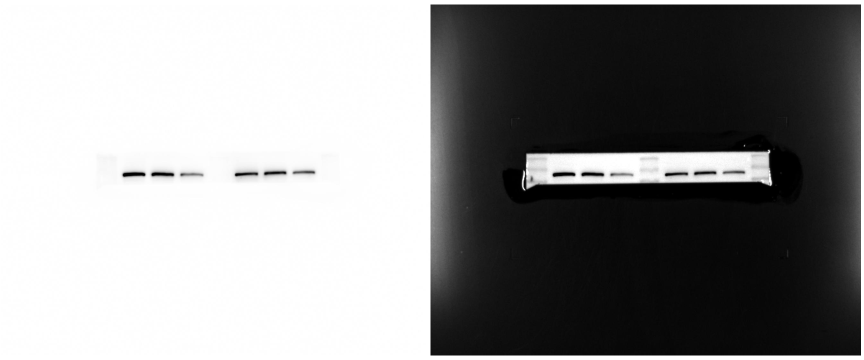

β-actin

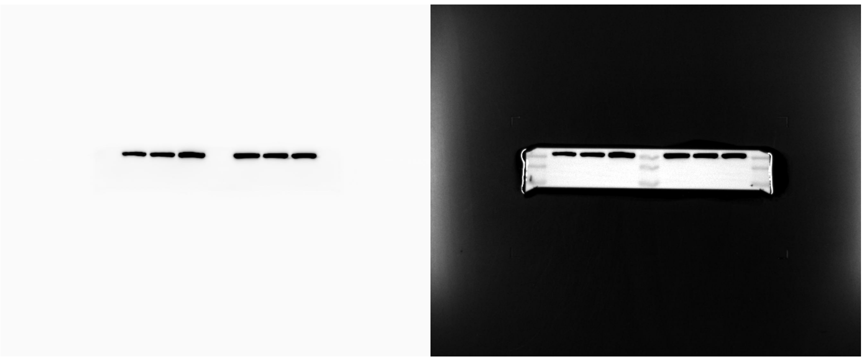

Figure 7B

OVCAR-3

LC3

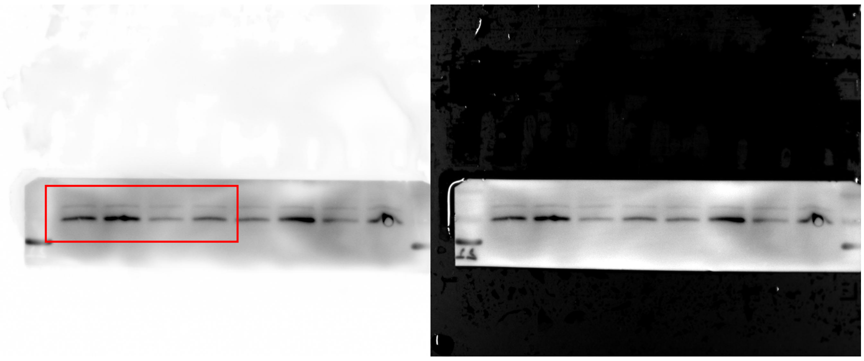

p62

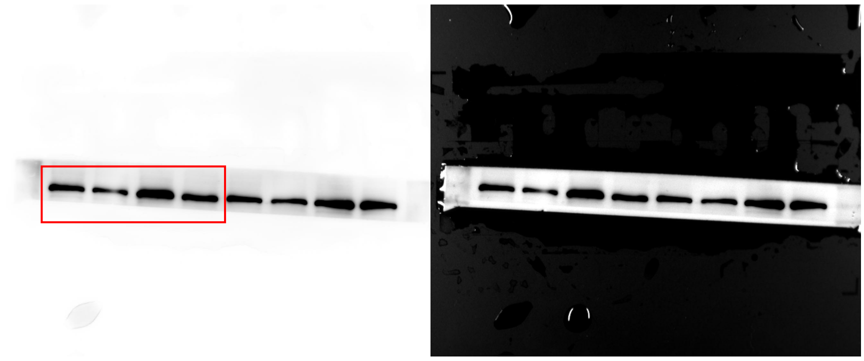

β-actin

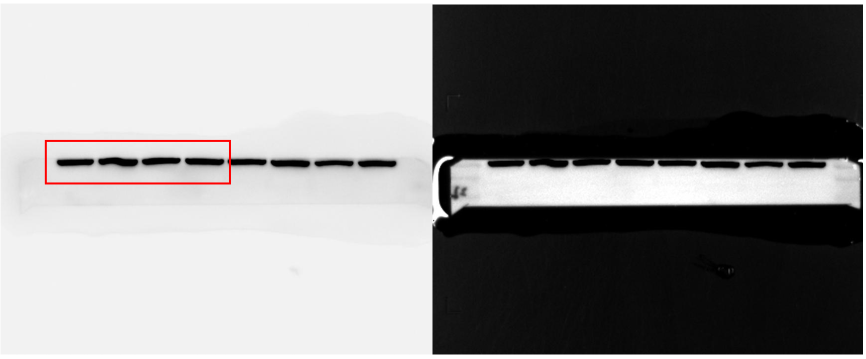

ES-2

LC3

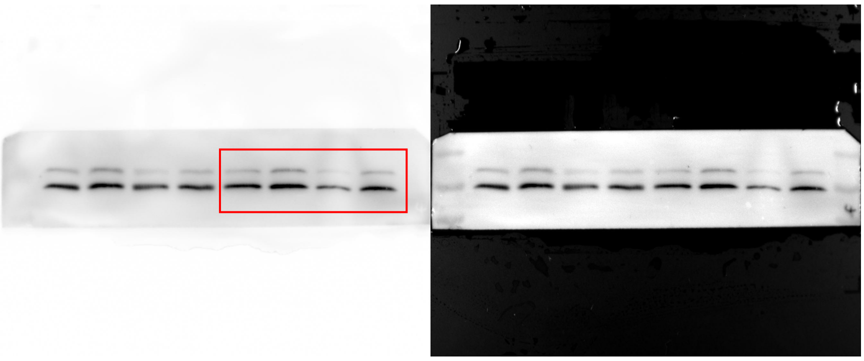

p62

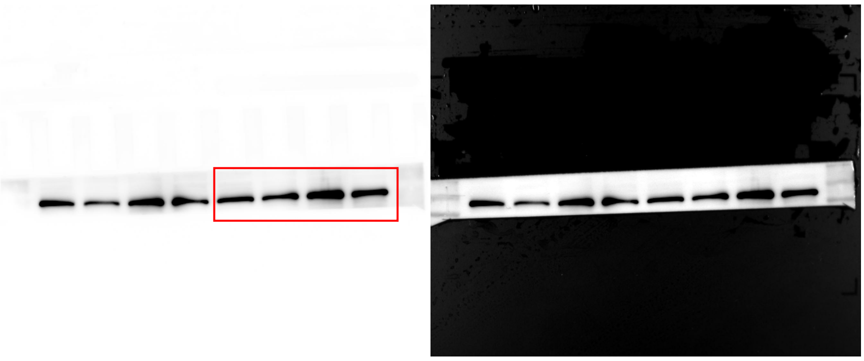

β-actin

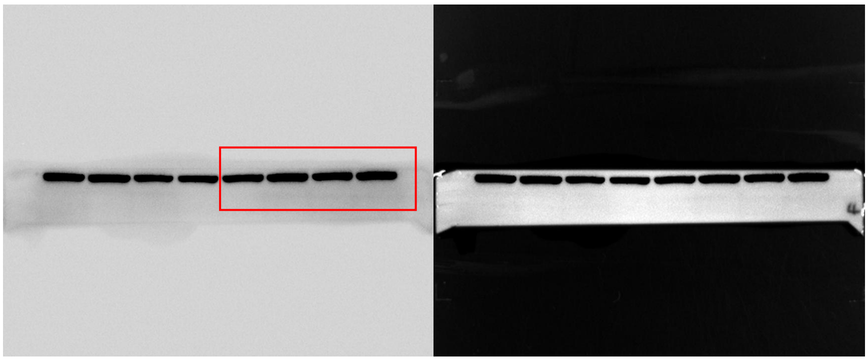

Figure S3E

P53

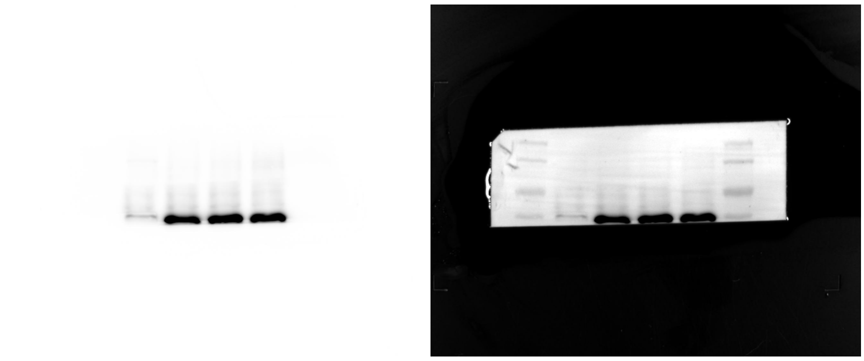

β-actin

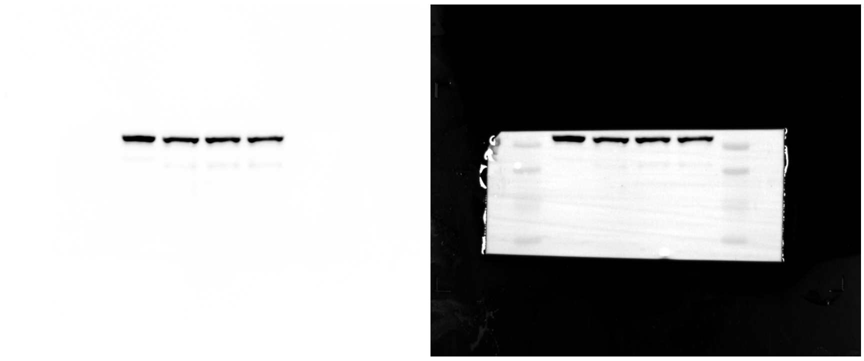

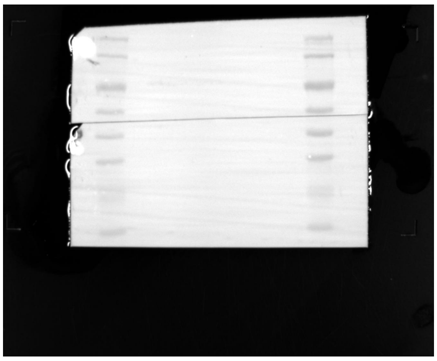

Figure S3F

WTAP

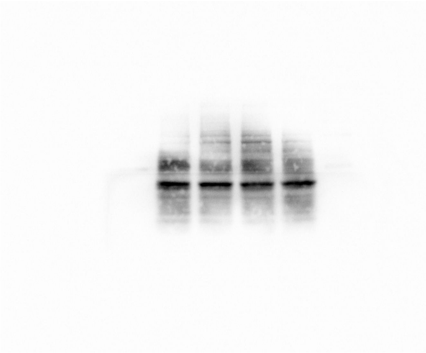

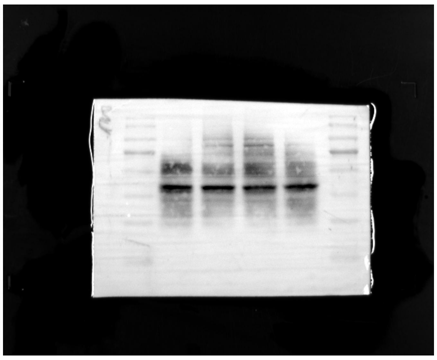

β-actin

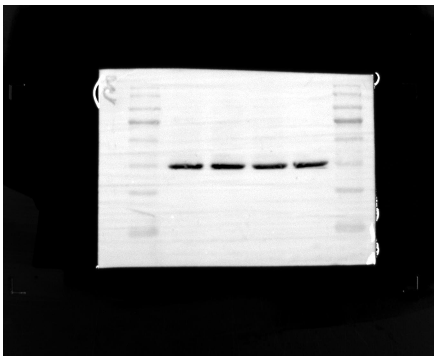

Supplement: Supplementary file 8 — Original Data File [file 41419_2024_6477_MOESM8_ESM.pdf]
